# COSA-1–SLX-4 interaction directly links crossover designation with Holliday junction resolution

**DOI:** 10.1126/sciadv.adx9148

**Published:** 2026-04-24

**Authors:** Guoteng Liu, Yuejun Yang, Wencong Nan, Tongxin Xiao, Zongyu Guo, Meiyu Zhang, Yuchen Wang, Xuezhen Wu, Anton Gartner, Hongtao Zhang, Ye Hong

**Affiliations:** ^1^ Shandong Provincial Key Laboratory of Development and Regeneration, School of Life Sciences, Shandong University, Qingdao, Shandong 266237, China.; ^2^ Department of Pathology, Shandong Cancer Hospital and Institute, Shandong First Medical University and Shandong Academy of Medical Sciences, Jinan, Shandong 250117, China.; ^3^ Institute for Basic Sciences Center for Genomic Integrity, Graduate School for Health Sciences and Technology and Department for Biological Sciences, Ulsan National Institute of Science and Technology, Ulsan, Republic of Korea.; ^4^ Suzhou Research Institute of Shandong University, Suzhou, Jiangsu 215123, China.

## Abstract

Crossover (CO) formation between homologous chromosomes is essential for genetic diversity and accurate meiotic chromosome segregation. This process involves two key steps: the designation of a subset of meiotic double-strand breaks to CO-designated sites and subsequent CO resolution by Holliday junction (HJ) resolvase. However, how these steps are functionally coupled remains elusive. Here, we showed that COSA-1, essential for CO designation, directly interacts with the SLX-4 scaffold protein, which organizes the SLX-1, XPF-1, and MUS-81 HJ resolvases. Disrupting this interaction results in persistent unrepaired recombination intermediates and defective CO formation. Notably, these defects can be largely rescued by the artificial tethering of SLX-4 to the CO designation proteins. We further demonstrate that COSA-1 promotes assembly of the SLX-4 resolvase complex and provide evidence that this mechanism coupling CO designation with resolution is evolutionarily conserved. Together, our findings support a model in which CO designation proteins ensure accurate CO formation by directly recruiting the resolution machinery.

## Teaser

A conserved molecular recruitment process ensures accurate reciprocal exchanges between homologous chromosomes during meiosis.

## INTRODUCTION

Crossover (CO) formation between homologous chromosomes is essential for meiotic genetic exchange and accurate chromosome segregation in most sexually reproducing organisms. During meiotic prophase, COs are generated through repair by homologous recombination of programmed double-strand breaks (DSBs). In early meiotic cells, DSBs are induced enzymatically by the type II topoisomerase-like enzyme SPO-11 (*1*). DSB ends are then resected to produce single-stranded DNA (ssDNA), which can be bound by RAD-51 recombinase to promote strand invasion into homologous duplex DNA. The invading strand can be processed by two distinct pathways: the double Holliday junction (dHJ) pathway, where second-end capture enables the formation of dHJs that are resolved predominantly into COs, and the synthesis-dependent strand annealing (SDSA) pathway, where helicase-mediated displacement of the invading strand enables annealing to the other DSB end, generating exclusively noncrossover (NCO) products (*2*).

The preference for either of these two distinct pathways indicates that the outcome of DSB repair is precisely regulated. Despite numerous DSBs forming, only a subset is channeled toward CO formation (*2*). This selective process, also known as CO designation, is orchestrated by CO designation factors, a group of specialized proteins that recognize and stabilize recombination intermediates between homologous chromosomes (*2*–*4*). Key CO designation factors include the ZMM protein group (Zip1-Zip4, Msh4-Msh5, and Mer3 in budding yeast) (*5*). In budding yeast, most recombination intermediates stabilized by ZMMs mature into COs (*3*, *5*). However, ZMM foci exceed CO numbers, indicating that ZMM-bound recombination intermediates are not exclusively committed to CO outcomes. *Caenorhabditis elegans* exhibits strict ZMM-dependent CO regulation, with all COs being processed through this pathway (*6*). In *C. elegans*, MSH-5 foci initially appear abundantly during early pachytene, likely marking multiple recombination intermediates (*7*). By late pachytene, however, these foci consolidate into a single large focus per homolog pair, forming a distinctive complex containing COSA-1 and ZHP-3/4 as well (*8*). This singular focus designates the sole CO event for each pair of chromosomes during *C. elegans* meiosis.

To achieve the obligate CO, CO designation must be followed by efficient resolution. CO resolution occurs through cleavage of HJs by structure-selective endonucleases, which primarily generate CO products during meiosis (*9*). In budding yeast and mouse, the resolution of HJs requires two conserved endonuclease activities: the MutLγ complex (MLH1-MLH3 heterodimer) and the MUS81–EME1 endonuclease (Mus81-Mms4 in yeast) (*10*–*13*). In *C. elegans*, genetic studies have defined two parallel, partially redundant HJ resolution pathways that contribute to CO formation: one dependent on the XPF-1 endonuclease and the *C. elegans* Bloom helicase ortholog HIM-6 and the other dependent on SLX-1 and MUS-81 nucleases (*14*–*18*). Mutations in individual components of these pathways cause only minor defects in CO formation on their own, consistent with their redundant roles. Both pathways require the SLX-4 scaffold, which functions as a large scaffold protein containing multiple domains that recruit XPF-1-ERCC-1, SLX-1, and MUS-81 nucleases (*19*).

CO designation and resolution represent distinct mechanistic steps during CO formation. However, the initial trigger for recruiting resolvases to CO-designated sites has remained elusive. Our previous study showed that the key CO designation protein COSA-1 interacts with other CO designation factors to assemble functional “recombination nodules” at CO-designated sites (*20*). This complex prevents early CO-designated recombination intermediates from being dismantled by the RTEL-1 helicase and protects late recombination intermediates until they are resolved. Here, we show that COSA-1 directly recruits HJ resolvases via SLX-4 to orchestrate CO designation and resolution. We provide evidence that the interaction between CO designation factors and resolvases is conserved in vertebrates.

## RESULTS

### *C. elegans* SLX-4 physically interacts with COSA-1

**Fig. 1.**
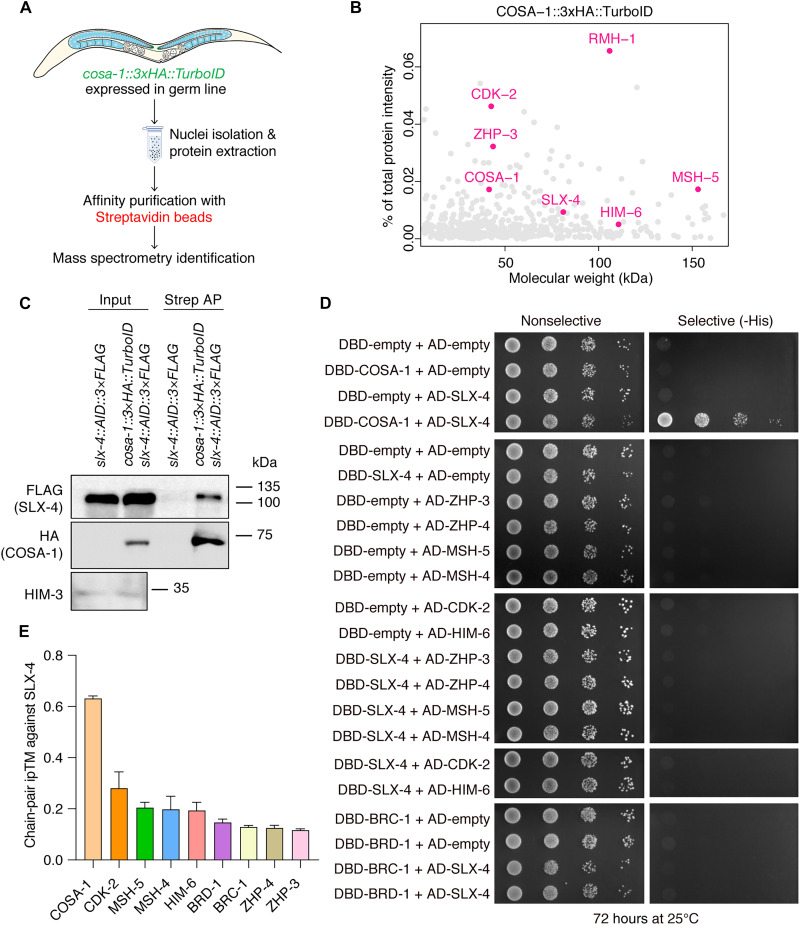
*C. elegans* SLX-4 physically interacts with COSA-1. (**A**) Diagram of the identification of interactors of COSA-1 using TurboID-mediated proximity labeling. (**B**) The relative abundance of proteins labeled by COSA-1::3×HA::TurboID-mediated biotinylation and quantified by MS is defined as their intensity percentage relative to the total protein intensity (*y* axis), with values arranged in order of molecular weight (*x* axis). Pink points indicate proteins detected in all three replicates, with the remaining proteins depicted in gray. (**C**) Biotinylated SLX-4 was detected by Western blotting with antibody against FLAG tag. (**D**) Analysis of interactions between COSA-1 and SLX-4 by yeast two-hybrid assays. (**E**) Bar graph showing the chain-pair ipTM for SLX-4 against each CO designation protein predicted by AlphaFold. A higher ipTM value indicates a stronger predicted interaction. Sample size, *n* = 10 for each group.

We performed TurboID-MS analysis to identify proteins closely associated with COSA-1 (Fig. 1A) (*20*). Prominent candidates included the known CO designation factors CDK-2, ZHP-3, and RMH-1 and, unexpectedly, the SLX-4 scaffolding protein (Fig. 1B). TurboID-coupled Western blotting confirmed that SLX-4 is associated with COSA-1 (Fig. 1C). Analysis of biotinylation signal localization using the *cosa-1::3×HA::TurboID* strain revealed that the signal is absent in the mitotic progenitor zone, emerges upon meiotic entry, and intensifies throughout pachytene, indicating that the association of COSA-1 with its partners, including SLX-4, is meiosis specific (fig. S1). Yeast two-hybrid assays demonstrated that SLX-4 physically interacts with COSA-1 but not with other CO designation factors (ZHP-3/4, MSH-4/5, CDK-2, and HIM-6) (Fig. 1D). To further investigate the SLX-4–COSA-1 interaction, we performed protein-protein interaction predictions using AlphaFold. Notably, the SLX-4–COSA-1 complex displayed the highest interface predicted template modeling (ipTM) score among known CO designation factors (Fig. 1E).

**Fig. 2.**
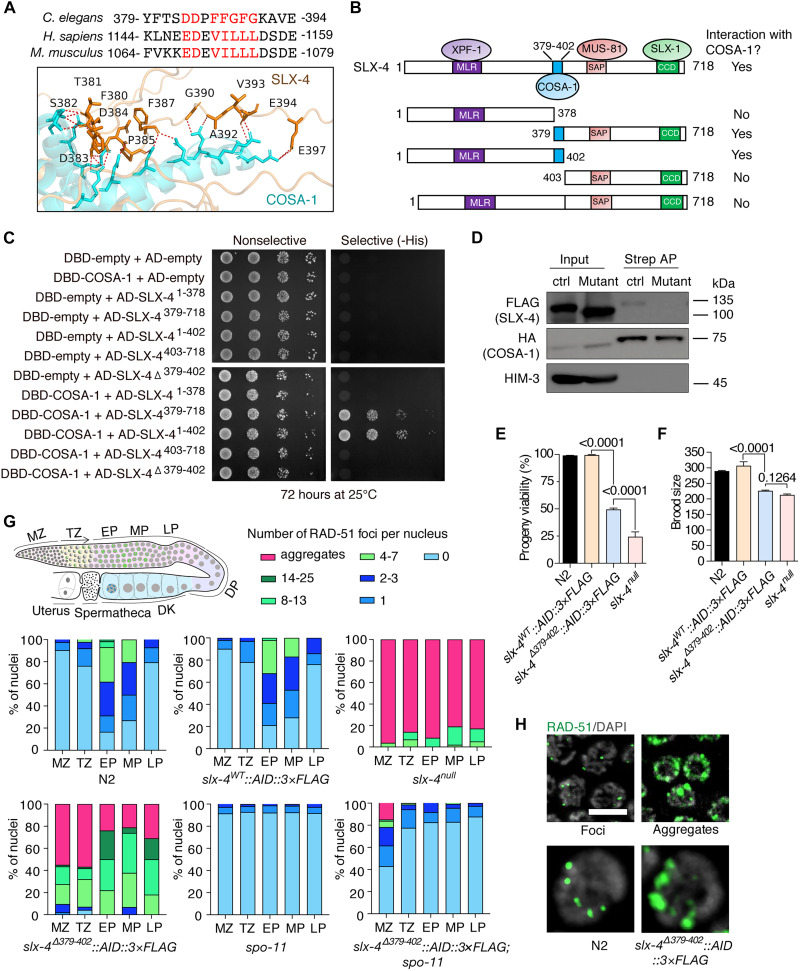
The COSA-1 binding region in SLX-4 coordinates DSB repair. (**A**) Schematic illustration of the COSA-1 interaction region on SLX-4. The structure of COSA-1–SLX-4 complex was predicted by AlphaFold 3. The amino acid residues on SLX-4 that are potentially involved in the interaction are highlighted in red. (**B**) Schematic illustration of the interaction between COSA-1 and different SLX-4 truncated mutants. (**C**) Analysis of the interaction between COSA-1 and different SLX-4 truncated mutants by yeast two-hybrid assays. (**D**) Detection of the SLX-4^Δ379–402^ mutant protein in biotinylated proteins purified from whole-worm lysates of *cosa-1::3×HA::TurboID slx-4*^Δ^*^379–402^::3×FLAG* strain. Ctrl: *cosa-1::3×HA::TurboID slx-4::3×FLAG*; Mutant: *cosa-1::3×HA::TurboID slx-4*^Δ^*^379–402^::3×FLAG*. (**E**) Progeny viability of the indicated genotypes. (**F**) Brood size of the indicated genotypes. (**G**) Quantification of RAD-51 foci in indicated regions of the germ line. MZ, mitotic zone; TZ, transition zone; EP, early pachytene; MP, mid-pachytene; LP, late pachytene. (**H**) Representative images of nuclei with RAD-51 foci or aggregates. The left panel (showing RAD-51 foci) is from wild-type early pachytene nuclei, and the right panel (showing aggregates) is from *slx-4*^Δ^*^379–402^::AID::3×FLAG* late pachytene nuclei. Scale bar, 5 μm.

On the basis of the AlphaFold prediction (Fig. 2A), we next used a series of SLX-4 truncations in yeast two-hybrid assays to experimentally confirm the precise region that interacts with COSA-1. Consistent with structural modeling, COSA-1 specifically bound to the flexible loop spanning residues 379 to 402 but showed no detectable affinity for either the N- or C-terminal regions (Fig. 2, B and C). Notably, deletion of residues 379 to 402 (SLX-4^Δ379–402^) did not disrupt SLX-4’s known interactions with XPF-1 (via the MLR domain), MUS-81 (SAP domain), or SLX-1 (SBD domain) (fig. S2), indicating that this segment specifically contributes to COSA-1 binding. To validate this interaction in vivo, we generated a *cosa-1::3×HA::TurboID slx-4*^Δ^*^379–402^::3×FLAG* strain and performed Streptavidin affinity purification targeting biotinylated proteins from whole-worm lysates. Analysis of the purified fraction failed to detect the SLX-4^Δ379–402^ mutant protein, demonstrating that the 379 to 402 region is necessary for the interaction with COSA-1 (Fig. 2D).

Having established the central SLX-4 loop (amino acids 379 to 402) as the specific binding interface for COSA-1 in vitro, we next investigated its functional role during meiotic recombination in vivo. Using Cas9-mediated dual single guide RNA (sgRNA) excision, we generated an endogenous *slx-4*^^Δ^*379*–*402*^*::AID::3×FLAG* strain. The COSA-1 interaction domain deletion did not affect the localization of SLX-4. Immunostaining revealed a strong SLX-4 signal in both the mitotic zone and late pachytene, consistent with the wild-type pattern (fig. S3, A and C). As previously reported, the signal intensity was markedly reduced in the transition zone and in early and mid-pachytene stages (fig. S3, B and D) (*17*). These results confirm that the COSA-1 binding region is dispensable for the in vivo localization of SLX-4. However, the *slx-4*^Δ*379*–*402*^*::AID::3×FLAG* mutant exhibited substantially reduced brood size and embryonic viability compared to wild type, although less severe than the *slx-4 (tm2181)* null allele (Fig. 2, E and F, and table S1) (*17*).

To investigate whether the decreased brood size and viability in *slx-4*^Δ^*^379–402^::AID::3×FLAG* mutants stem from DSB repair defects, we analyzed RAD-51 recombinase dynamics during meiotic prophase (Fig. 2G). In wild type, RAD-51, which marks nascent DSBs, initially localize to discrete nuclear foci in the transition zone. The foci number peak in early pachytene nuclei and progressively decline through mid-pachytene. RAD-51 foci almost disappear by late pachytene, indicating efficient DSB repair. In contrast, both *slx-4* null and *slx-4*^Δ^*^379–402^::AID::3×FLAG* mutants displayed elevated RAD-51 focus numbers in mitotic and meiotic germ lines compared to wild type. Consistent with a previous study (*17*), large RAD-51 foci were present in both mutants. Notably, we additionally observed RAD-51 aggregates (Fig. 2H), which are morphologically distinct from discrete foci and not previously reported in *slx-4*–deficient backgrounds, in both *slx-4* null and *slx-4*^Δ^*^379–402^::AID::3×FLAG* mutants. To determine whether the RAD-51 aggregates originate from DNA lesions carried over from the mitotic progenitor zone, we depleted SLX-4 using an auxin-inducible degron system. As shown in fig. S4, RAD-51 aggregates were already observed in mid-pachytene nuclei after 12 hours of depletion. On the basis of the established progression rate of approximately one row per 1.1 hours in the *C. elegans* gonad, a 12-hour period is insufficient for nuclei that were in the mitotic progenitor zone at the start of depletion to have reached mid-pachytene (*21*, *22*). Therefore, the RAD-51 aggregates observed at mid-pachytene after 12 hours of SLX-4 depletion must correspond to nuclei that were already committed to meiosis and in early prophase at the time of depletion. This indicates that the DNA lesions marked by these aggregates are generated de novo during meiotic prophase, rather than being imported from the mitotic region. In addition, we found that these persistent RAD-51 foci and aggregates were virtually abolished in meiotic nuclei of the *slx-4*^Δ^*^379–402^::AID::3×FL*AG; *spo-11* mutant, indicating that their formation largely depends on SPO-11–generated meiotic DSBs (Fig. 2G). Together, these data demonstrate that the SLX-4–COSA-1–interacting domain plays a role in efficient DSB repair during mitosis and meiotic prophase. In the following sections, we showed that the same domain also performs an independent and separate function by mediating the SLX-4–COSA-1 interaction to ensure CO resolution.

### The interaction between SLX-4 and COSA-1 is required for CO intermediate resolution

**Fig. 3.**
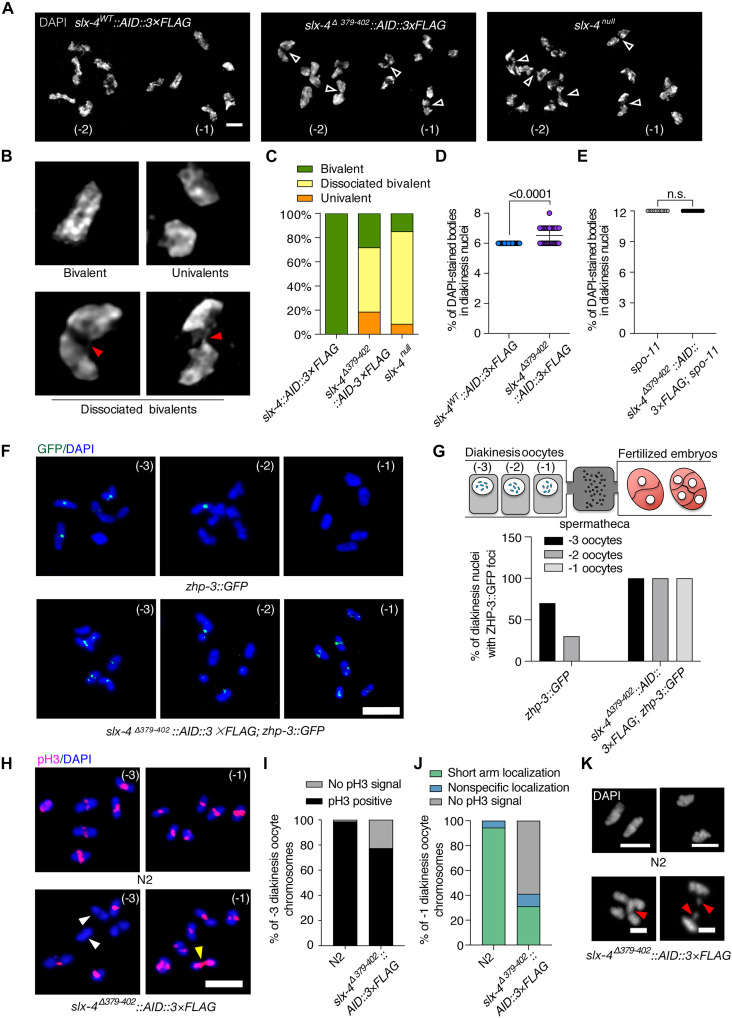
*slx-4*^Δ*379–402*^ mutant is defective in CO formation. (**A**) DAPI-stained diakinesis chromosomes taken by super-resolution SIM. Open arrowheads indicate the linked chromosomes. Scale bar, 2 μm. (**B**) Images of representative chromosomes from bivalents, univalents, and dissociated bivalents. Red arrowheads indicate DNA linkages. (**C**) Percentage of the bivalents, dissociated bivalents, and univalents for each genotype. (**D** and **E**) Quantitative analysis of the number of DAPI-stained bodies in diakinesis nuclei in *slx-4*^Δ*379–402*^ and *slx-4*^Δ^*^379–402^::AID::3×Flag; spo-11* mutants. n.s., not significant. (**F**) Immunofluorescence images of diakinesis (-1, -2, and -3) nuclei stained for DAPI (blue) and ZHP-3::GFP (green). Scale bar, 5 μm. (**G**) Quantification of the percentage of diakinesis nuclei with ZHP-3::GFP foci. (**H**) Immunostaining for Ser^10^-phosphorylated histone H3 (Ser^10^ pH3; red) and DAPI (blue) in diakinesis (-1 and -3) nuclei. Scale bar, 5 μm. White arrowheads indicate bivalents with no pH3 signal. Yellow arrowheads indicate bivalents with nonspecific localization of pH3. (**I** and **J**) Quantification of bivalents with different pH3 localization patterns in diakinesis (-1 and -3). (**K**) Chromosome segregation in wild type and the *slx-4*^Δ*379–402*^ mutant during anaphase I. Red arrowheads indicate chromatin bridges or lagging chromosomes. Chromatin was stained with DAPI (white). Scale bars, 2 μm.

We sought to specifically investigate the function of the SLX-4–COSA-1 interaction during meiotic prophase. Defective conversion of recombination intermediates to mature COs is often accompanied by elevated RAD-51 levels and its delayed removal during meiotic prophase (*14*–*17*). Given the persistence of RAD-51 foci in *slx-4*^Δ^*^379–402^::AID::3×FLAG* mutants, we first investigated whether compromised resolution of recombination intermediates impairs CO formation. To assess CO formation, we examined the morphology and number of diakinesis chromosomes. In wild type, six compact bivalents can be observed in the last two oocytes before fertilization (-1 and -2 oocytes; Fig. 3, A to D). In contrast, super-resolution structured illumination microscopy (SIM) revealed aberrant chromosome associations in mutants: ~53% of 4′,6-diamidino-2-phenylindole (DAPI)–stained bodies exhibited a “dissociated bivalent” structure (Fig. 3, B and C), where homologs remained loosely connected by DNA bridges. This morphology, characteristic of unresolved recombination intermediates (*14*), indicated compromised CO formation. Critically, these DNA bridges were abolished in *slx-4*^Δ^*^379–402^::AID::3×FLAG; spo-11* double mutants, which consistently exhibited 12 univalents (Fig. 3E), confirming that the aberrant connections are SPO-11 dependent and thus originate from unresolved meiotic recombination intermediates.

Previous studies have shown that unresolved recombination intermediates cause delayed dissociation of CO designation proteins from CO designation sites in diakinesis oocytes (*17*, *20*). To determine whether the dissociation of CO designation factors from chromosomes was also affected in the *slx-4*^Δ^*^379–402^::AID::3×FLAG* mutant, we analyzed the dynamics of ZHP-3, a CO designation marker previously used to monitor the dissociation of CO factors in diakinesis (*17*, *20*). In late pachytene, both wild-type and *slx-4*^Δ^*^379–402^::AID::3×FLAG* nuclei formed six distinct ZHP-3 foci, indicating that CO designation remains intact in the mutant (fig. S5). However, the subsequent dissociation of ZHP-3 was significantly delayed in the mutant. During normal meiotic progression to diakinesis, CO designation proteins gradually dissociate from CO sites, becoming undetectable in most -3 and -2 oocytes and completely absent in all -1 diakinesis oocytes (Fig. 3, F and G) (*14*). In contrast, persistent chromosome-associated ZHP-3 foci could be detected in -1 diakinesis oocytes in the *slx-4*^Δ^*^379–402^::AID::3×FLAG* mutants (Fig. 3, F and G). This phenotype was not specific to ZHP-3. We observed similar persistence of other CO designation factors on diakinesis chromosomes in the *slx-4*^Δ^*^379–402^::AID::3×FLAG* mutants. Both MSH-5 and COSA-1 formed normal late pachytene foci but subsequently failed to dissociate in a timely manner (fig. S6). The persistent association of multiple CO designation factors collectively implies defective processing of CO intermediates in the mutants.

Mature CO formation is required for the timely localization of phosphorylated histone H3 (pH3) to the short arms of bivalents (*17*, *23*). Therefore, we used pH3 as a marker to further assess bivalent maturation in our mutants. In wild-type oocytes, the pH3 signal was robust and specifically enriched on the short arms. However, in the *slx-4*^Δ*379–402*^ mutant, pH3 localization was both delayed and aberrant: Only 77.4% of chromosomes in -3 oocytes were pH3 positive (compared to 98.1% in wild type) (Fig. 3, H and I); in addition, in -1 oocytes, 10% of chromosomes exhibited a nonspecific pH3 signal throughout the entire bivalents (compared to 5.6% in wild type) (Fig. 3, H and J).

Defective resolution of CO intermediates ultimately leads to chromosome segregation errors during meiosis I (*24*). To directly test whether the *slx-4*^Δ*379*–*402*^ mutation affects this critical step, we analyzed chromosome segregation in anaphase I oocytes. Chromatin bridges as well as lagging chromosomes were present in mutant oocytes but absent in wild-type controls (Fig. 3K), indicating defects in the resolution of physical connections. This phenotype, combined with the delayed RAD-51 removal, persistent CO designation factors, aberrant diakinesis bivalents, and defective pH3 localization, demonstrates that the SLX-4–COSA-1 interaction is essential for complete resolution of CO intermediates to ensure accurate chromosome segregation.

### Arg^163^ in COSA-1 mediates SLX-4 binding to promote CO resolution

**Fig. 4.**
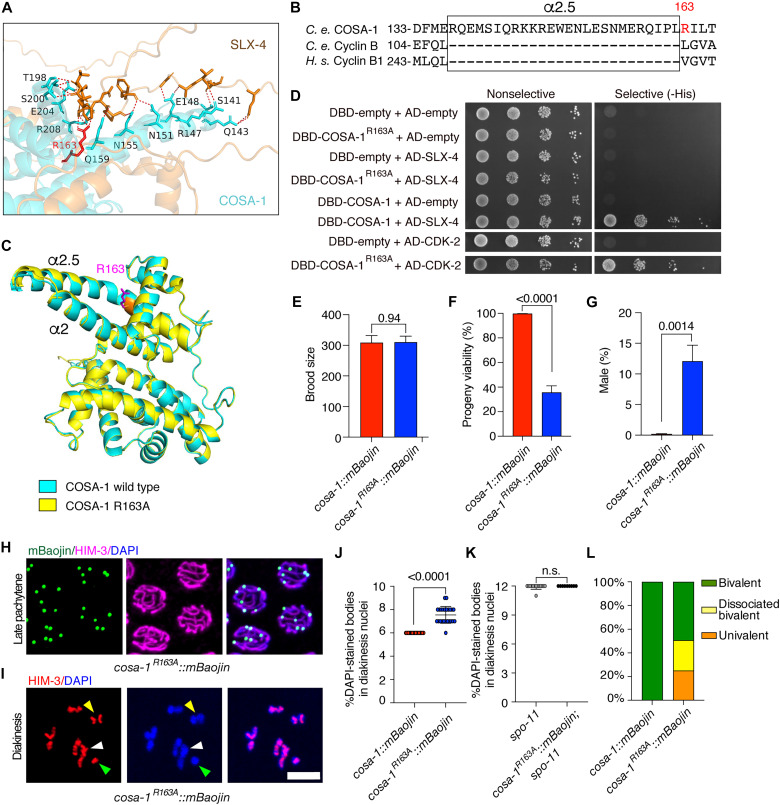
Arg^163^ in COSA-1 mediates SLX-4 binding to promote CO resolution. (**A**) Interaction interface between SLX-4 and COSA-1 predicted by AlphaFold. Potential SLX-4–binding residues within the COSA-1 structure are shown. (**B**) Sequence alignment among *C. elegans* COSA-1, Cyclin B, and human Cyclin B1. The additional helix (α2.5) presented only in COSA-1 and the key residue R163 involved in the interaction are indicated. (**C**) Structural comparison between the wild-type COSA-1 and COSA-1^R163A^ mutant. Both structures were generated by AlphaFold. (**D**) Analysis of the interaction between COSA-1^R163A^ and SLX-4 or CDK-2 by the yeast two-hybrid assay. (**E** to **G**) Quantification of the brood size, progeny viability, and frequencies of male offspring among the progeny of the indicated genotypes. (**H**) Representative images of late pachytene nuclei with COSA-1 foci of *cosa-1^R163A^::mBaojin*. Scale bar, 5 μm. (**I**) Representative images of diakinesis nuclei of *cosa-1^R163A^::mBaojin* stained with antibodies against HIM-3 (red), counterstained with DAPI (blue). Scale bar, 5 μm. (**J** and **K**) Quantitative analysis of the number of DAPI-stained bodies in diakinesis nuclei for each genotype. (**L**) Percentage of the bivalents, dissociated bivalents, and univalents for each genotype.

To further dissect the interaction between SLX-4 and COSA-1 and to corroborate the importance for this for HJ resolution, we carefully examined their AlphaFold-predicted complex structure, which revealed that the N-terminal cyclin box domain of COSA-1 mediates the interaction with SLX-4 (Fig. 4A). Notably, this cyclin box domain harbors a unique α helix (α2.5) exclusive to the COSA-1/CNTD1 protein family, structurally distinguishing it from canonical cyclins (Fig. 4, B and C). To validate this interface, we introduced targeted mutations in COSA-1 at residues computationally predicted to mediate SLX-4 binding. Yeast two-hybrid assays demonstrated that substituting Arg^163^ with alanine (R163A) specifically abolished SLX-4 binding while preserving the interaction with CDK-2 kinase (Fig. 4D and fig. S7A).

We subsequently engineered the corresponding endogenous mBaojin-tagged *cosa-1^R163A^* allele via CRISPR-Cas9 to assess physiological relevance (*25*). Homozygous *cosa-1^R163A^::mBaojin* mutants exhibited wild-type brood size but displayed significantly reduced progeny viability and a pronounced high incidence of male progeny (Him phenotype; Fig. 4, E to G, and table S1). Intriguingly, despite these fertility defects, COSA-1^R163A^ retained normal spatiotemporal dynamics, with six distinct foci forming in late pachytene nuclei comparable to wild-type controls (Fig. 4H and fig. S7B).

To determine whether the disrupted SLX-4 interaction led to defective repair of meiotic DSBs, we examined RAD-51 focus formation. The *cosa-1^R163A^* mutants exhibited a significant increase in RAD-51 foci compared to wild type, and these foci persisted into late pachytene (fig. S7, C to E), indicating a failure to properly process recombination intermediates. This confirms that the R163A mutation, by specifically disrupting the COSA-1–SLX-4 interaction, impairs meiotic DSB repair.

Given that male progeny frequency inversely correlates with CO efficiency, we hypothesized that the observed high incidence of males in *cosa-1^R163A^* mutants stems from defective chiasmata formation. To test this, we examined chromosome morphology at diakinesis by simultaneously staining DNA with DAPI and meiotic chromosome axes with an antibody against HIM-3. Wild-type oocytes consistently displayed six condensed DAPI-stained bodies with a characteristic cruciform HIM-3 pattern at each chiasma site, confirming stable bivalent formation. Conversely, *cosa-1^R163A^* mutants displayed aberrant chromosome configurations, with an average of 7.5 ± 0.15 DAPI bodies (means ± SEM; *n* = 23 nuclei) (Fig. 4, I and J), indicating a mixture of univalents and bivalents. The cruciform HIM-3 pattern was also markedly decreased (Fig. 4, I and L), with homologs frequently appearing as “dissociated bivalents” (Fig. 4I). Crucially, in *cosa-1^R163A^::mBaojin; spo-11* double mutants, these connections were eliminated and 12 univalents were observed (Fig. 4K), proving that they are SPO-11–dependent unresolved recombination intermediates. Thus, these results showed that COSA-1 Arg^163^-mediated SLX-4 binding is essential for efficient CO resolution, corroborating our data on COSA-1 binding-defective SLX-4^Δ379–402^.

### Artificially tethering SLX-4^Δ379–402^ to the CO designation sites restores CO formation

**Fig. 5.**
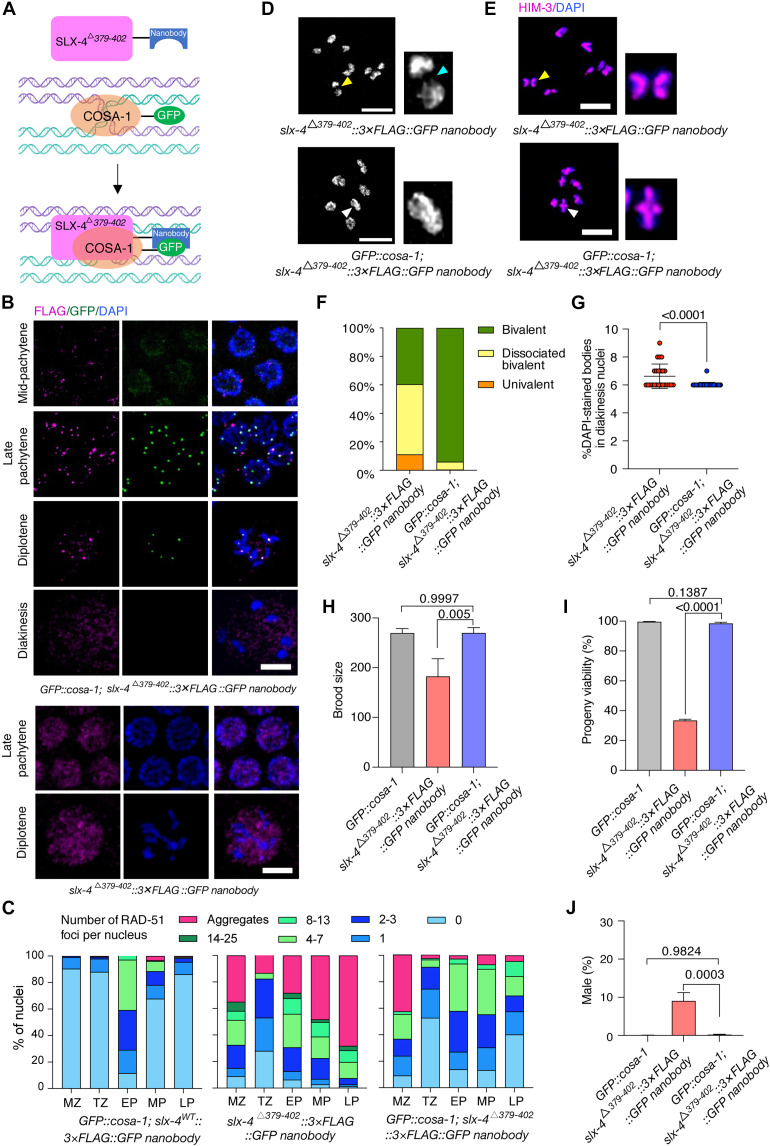
Artificially tethering interaction-compromised SLX-4 to GFP::COSA-1 restores CO formation. (**A**) Schematic illustration of the GFP nanobody–mediated interaction between SLX-4^Δ379–402^ and COSA-1. (**B**) Representative images of nuclei from indicated genotypes at various meiotic prophase stages, showing GFP–COSA-1 (green; detected with GFP-Booster), SLX-4 (magenta; detected with anti-FLAG), and DAPI (blue). Scale bars, 5 μm. (**C**) Quantification of RAD-51 foci in indicated regions of the germ line. MZ, mitotic zone; TZ, transition zone; EP, early pachytene; MP, mid-pachytene; LP, late pachytene. (**D**) Representative images of diakinesis nuclei of the indicated genotypes stained with antibodies against HIM-3 (magenta), counterstained with DAPI (blue). Dissociated bivalent in the *slx-4*^Δ*379–402*^ mutant and bivalent with cruciform HIM-3 patterning in *GFP::cosa-1; slx-4*^Δ^*^379–402^::3×FLAG::GFP nanobody* are indicated by yellow and white arrowheads, respectively, and shown in close-ups. Scale bars, 5 μm. (**E**) DAPI-stained diakinesis chromosomes taken by super-resolution SIM. Yellow and cyan arrowheads indicate dissociated bivalent and DNA linkages, respectively. Scale bars, 5 μm. (**F**) Percentage of the bivalents, dissociated bivalents, and univalents for each genotype. (**G**) Quantitative analysis of the DAPI-stained bodies in diakinesis nuclei of each genotype. (**H** to **J**) Quantification of the brood size, progeny viability, and frequencies of male offspring among the progeny of the indicated genotypes.

To functionally validate that the SLX-4–COSA-1 interaction directly drives CO formation, we artificially restored this interaction in *slx-4*^Δ*379–402*^ mutants using a green fluorescent protein (GFP) nanobody tethering strategy (Fig. 5A). We generated a *slx-4*^Δ*379–402*^::3×FLAG ::GFP nanobody fusion worm strain. In this strain, COSA-1 formed normal foci in late pachytene but exhibited delayed dissociation at diakinesis (fig. S8), mirroring the defect seen in the *slx-4*^Δ*379*–*402*^*::AID::3×FLAG* mutant. The SLX-4^Δ379–402^ protein in these mutants displayed a diffuse nuclear localization during late pachytene and diplotene (Fig. 5B). Notably, when crossed with GFP-tagged COSA-1 strains, SLX-4^Δ379–402^ was robustly recruited to chromosomes, forming discrete foci that predominantly colocalized with COSA-1 at designated CO sites (Fig. 5B). This forced spatial targeting evidently rescued the aberrant accumulation of RAD-51 foci in the meiotic region of the germ line but not that in the mitotic region (Fig. 5C and fig. S9), indicating that the rescue was specific for meiotic recombination defects. To rule out nonspecific effects of the artificial tethering strategy itself, we examined the control strain *GFP::cosa-1; slx-4^WT^::3×FLAG::GFP nanobody*. Its RAD-51 foci levels were comparable to wild type (Fig. 5C), indicating that the GFP nanobody–mediated tethering per se does not interfere with the function of wild-type SLX-4. Notably, bypassing the SLX-4^Δ379–402^ interaction defect also rescued defective chromosome morphogenesis: Dissociated bivalents connected by DNA bridges decreased from 49.3% to near wild-type levels (~5.5%, *P* < 0.0001) (Fig. 5, D and F), whereas stable chiasmata marked by cruciform HIM-3 structures were robustly restored in diakinesis oocytes (Fig. 5, E and G). Consequently, targeted recruitment of SLX-4^Δ379–402^ to GFP::COSA-1 fully reversed the fertility defects: Brood size and progeny viability returned to wild-type levels (Fig. 5, H and I). Furthermore, chromosome missegregation defects, specifically measured by the elevated Him phenotype, were abolished (Fig. 5J). These results demonstrate that proper recruitment of SLX-4 to COSA-1 is critical for efficient CO formation.

**Fig. 6.**
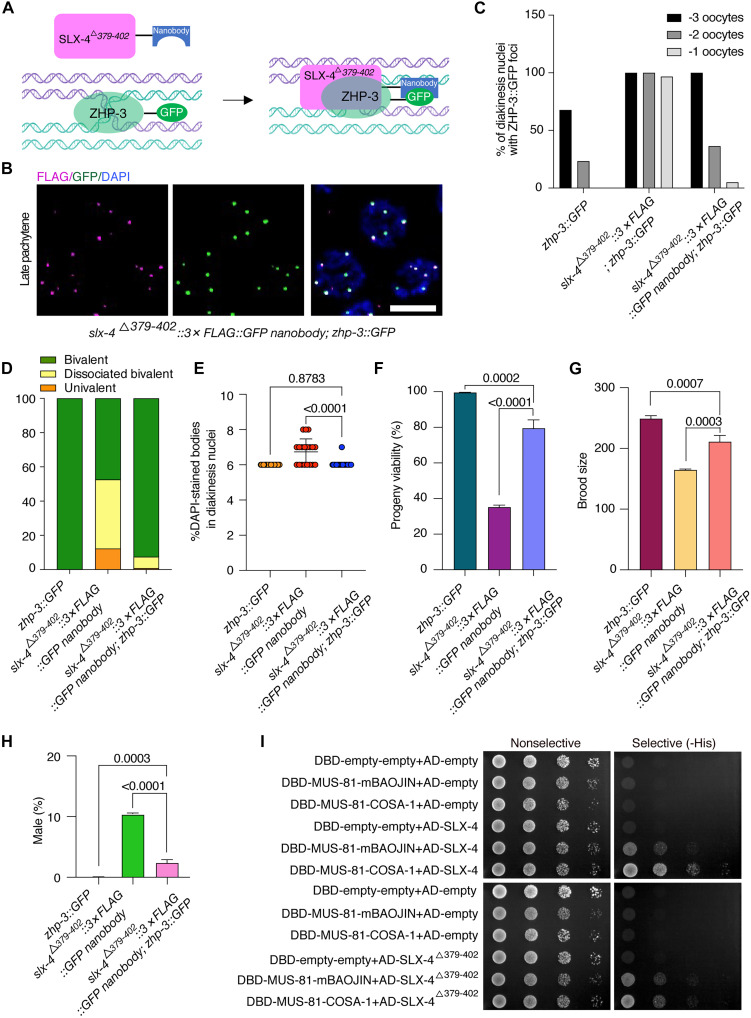
Tethering SLX-4^Δ379–402^ to ZHP-3::GFP partially rescued the meiotic defects. (**A**) Schematic illustration of the GFP nanobody–mediated interaction between SLX-4^Δ379–402^ and ZHP-3. (**B**) Representative images of late pachytene nuclei of the indicated genotypes stained with GFP-Booster to detect ZHP-3::GFP (green) and antibodies against FLAG (magenta) to detect SLX-4, counterstained with DAPI (blue). Scale bar, 5 μm. (**C**) Quantification of the percentage of diakinesis nuclei with ZHP-3::GFP foci. (**D**) Percentage of the bivalents, dissociated bivalents, and univalents for each genotype. (**E**) Quantitative analysis of the number of DAPI-stained bodies in diakinesis nuclei of each genotype. (**F** to **H**) Quantification of the brood size, progeny viability, and frequencies of male offspring among the progeny of the indicated genotypes. (**I**) Analysis of the effect of COSA-1 binding on the SLX-4–MUS-81 interaction by yeast three-hybrid assays.

To further determine whether recruitment to CO-designated sites is sufficient for SLX-4 function irrespective of specific interactions with COSA-1, we used the GFP-targeted nanobody approach in a *zhp-3::GFP* genetic background (Fig. 6A). We found that the interaction-deficient mutant SLX-4^Δ379–402^ was robustly localized to ZHP-3–enriched CO designation sites (Fig. 6B). Crucially, artificial tethering of SLX-4^Δ379–402^ to ZHP-3::GFP significantly rescued major meiotic defects in *slx-4*^Δ*379–402*^ mutants (Fig. 6, C to H). The delayed dissociation of ZHP-3 from CO designation sites in diakinesis oocytes was largely rescued, with only 5% of -1 oocytes examined in *slx-4*^Δ^*^379–402^::3×FLAG::GFP nanobody; zhp-3::GFP* worms showing persistent ZHP-3 foci versus 96.7% in the *slx-4*^Δ^*^379–402^::3×FLAG; zhp-3::GFP* mutant (Fig. 6C). In addition, the incidence of dissociated bivalents was decreased by sixfold (from 40.35 to 6.67%), and the number of DAPI-stained bodies in mature oocytes was reduced to the wild-type level (Fig. 6, D and E). Furthermore, the brood size was recovered from 65.9 to 84.7% of wild-type levels, the progeny viability reached 79.5% (35.2% in *slx-4*^Δ^*^379–402^::3×FLAG::GFP nanobody* mutant), and chromosome segregation fidelity was normalized as evidenced by suppression of the Him phenotype from 10.3 to 2.36% (*P* < 0.0001) (Fig. 6, F to H). Notably, the rescue efficiency mediated by ZHP-3 recruitment was consistently attenuated relative to COSA-1 tethering outcomes, particularly evident in progeny viability (79.5% versus 98.5%). This partial rescue demonstrates that SLX-4 function can be partially restored by forced localization to the CO designation sites.

Given that SLX-4 functions as an essential scaffold for HJ resolution nucleases and that COSA-1 binds to a specific region of SLX-4, we next asked whether this interaction influences SLX-4’s association with its partner endonucleases. Using yeast three-hybrid assays, we observed that the COSA-1–SLX-4 interaction significantly enhances SLX-4 binding to MUS-81 and XPF-1 (Fig. 6I and fig. S10A). This enhancement was abolished when using SLX-4^Δ379–402^ mutants (Fig. 6I). Similarly, although SLX-1 already exhibited strong basal binding to SLX-4, a modest but reproducible further increase was also detected in the presence of COSA-1, although the effect was less pronounced compared to MUS-81 and XPF-1 (fig. S10A). Because yeast two-hybrid assays detected no direct interaction between COSA-1 and MUS-81, XPF-1, or SLX-1 (fig. S10B), these results demonstrate that the COSA-1–SLX-4 interaction is essential for promoting nuclease binding. Collectively, these findings suggest that COSA-1 not only recruits SLX-4 to CO designation sites but also promotes its assembly with key endonucleases.

### Conservation of the CO designation factor-resolvase interaction

**Fig. 7.**
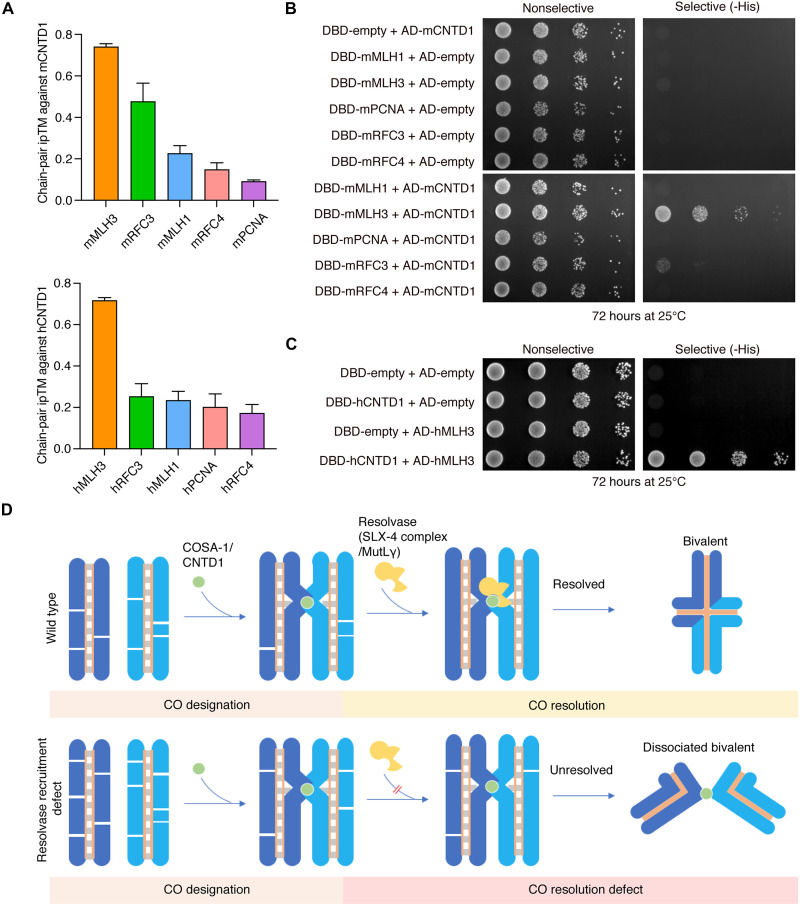
Conservation of the CO designation factor-resolvase interaction in mammals. (**A**) Bar graph showing the AlphaFold-predicted chain-pair ipTM between CNTD1 and MutLγ (along with its associated factors) in humans and mice. Sample size, *n* = 10 for each group. (**B**) Analysis of the interaction between mouse CNTD1 and MutLγ (along with its associated factors) by the yeast two-hybrid assays. (**C**) Analysis of the interaction between human CNTD1 and MLH3 by the yeast two-hybrid assays. (**D**) A cartoon model summarizes the interaction between CO designation factors and resolvases, which couples the processes of CO designation and resolution.

We next investigated whether the interaction between CO designation factors and resolvases is evolutionarily conserved. Given that meiotic HJ resolution in mammals is mediated by the MutLγ complex (MLH1-MLH3) (*26*), rather than SLX-4–associated nucleases used in *C. elegans*, we then focused on the MutLγ complex. Prior studies have shown that the endonuclease activity of MutLγ depends on activation by PCNA and the RFC loader complex (*12*, *13*). We therefore used structural modeling to predict interactions between CNTD1 and MutLγ, along with its regulatory factors. Notably, the predicted complex between MLH3 and CNTD1 yielded the highest ipTM score in both mice and humans (Fig. 7A). Using yeast two-hybrid assays with mouse proteins, we found that mCNTD1 strongly binds mMLH3 and weakly interacts with mRFC3 but shows no detectable binding to mMLH1, mPCNA, or mRFC4 (Fig. 7B). Moreover, we confirmed that this specific interaction between CNTD1 and MLH3 is conserved in humans (Fig. 7C). To define the interaction interface, we mapped the MLH3-binding region to the C terminus of CNTD1 (amino acids 181 to 334) (fig. S11A). Although a key arginine residue (R125 in mCNTD1 and R123 in hCNTD1) aligns with the SLX-4–interacting residue R163 in *C. elegans* COSA-1, it is located outside this binding region (fig. S11B). This indicates that, although a physical interaction between the designation factor and the resolvase is a conserved feature, the specific interaction motifs have diverged. Together, these results suggest that the recruitment of resolvase by CO designation factors represents a conserved functional coupling mechanism for CO formation across species.

## DISCUSSION

Our study revealed a specific interaction between the CO designation factor COSA-1 and a multidomain scaffold protein SLX-4. This interaction is essential for the recruitment of various endonucleases to CO designation sites, thereby directly coupling CO designation to resolution and ensuring proper CO formation (Fig. 7D).

Multiple evidence suggest that this mechanism may be widely conserved. First, we found that the mammalian ortholog of COSA-1, CNTD1, specifically interacts with the MutLγ resolvase subunit MLH3 (Fig. 7, B and C). Although additional functional studies in mammalian systems are needed, this physical interaction implies a potential role of CO designation factors in recruiting the resolvase machinery, similar to what we observed in *C. elegans*. Second, in species lacking a clear ortholog of COSA-1, such as budding yeast, the recombinase Dmc1, has been shown to physically associate with resolvase enzymes (*27*). This implies that the recruitment of resolvases by recombination factors may represent an ancient and conserved module within the meiotic recombination machinery.

Beyond recruitment, we provide evidence that the COSA-1–SLX-4 interaction promotes SLX-4 resolvase complex assembly. Whether this interaction also directly modulates the enzymatic activity of the complex remains an open question. However, several observations support a potential regulatory role. For instance, CNTD1 has been shown to interact with subunits of the RFC clamp loader complex, which loads PCNA. The PCNA-RFC complex strongly stimulates MutLγ endonuclease activity in vitro and is essential for MutLγ-dependent CO formation in vivo (*13*). In addition, in both yeast and humans, MutLγ interacts directly with MutSγ, leading to enhanced cleavage of HJs (*12*, *28*). Thus, physical interaction between CO designation factors and resolvases may represent a conserved mechanism across metazoans, and possibly beyond, that ensures spatiotemporal coordination and accuracy during meiotic CO formation.

Our work identified the specific binding interface mediating the COSA-1–SLX-4 interaction. We showed that mutation of a conserved arginine within the cyclin box domain of COSA-1 impairs its interaction with SLX-4. This arginine is located close to an extra predicted α helix occurring only in COSA-1 and distinguishes COSA-1 from conventional cyclins (*8*). We found that the small region on SLX-4, which interacts with COSA-1, encodes a putative SIM (SUMO-interaction motif). SIMs are hydrophobic peptide motifs that bind SUMO noncovalently, often exhibiting acidic flanking sequences. Human SLX-4 contains a cluster of three adjacent SIMs at this position, and these SIMs increase the interaction with specific DNA repair proteins and target SLX-4 to the sites of DNA damage (*18*, *29*, *30*). *C. elegans* contains only one SIM-like motif, which is located between the coiled-coil and SAP domains, similar to that of SLX-4 in humans. Although the SIM-like motif is likely to mediate the interaction with COSA-1 during meiotic recombination, it may also participate in recruiting other partners involved in DNA repair, consistent with excessive RAD-51 foci we observed in the mitotic region of the germ line. Nevertheless, our evidence that the very same domain specifically mediates the COSA-1 interaction, to bridge CO designation and SLX-4 complex–mediated HJ resolution during late prophase, is compelling. It is intriguing that artificially tethering interaction-compromised SLX-4^∆379–402^ with COSA-1 by the nanobody method can fully rescue the CO-deficient defects in SLX-4^Δ379–402^ mutants. Furthermore, this rescue effect was largely recapitulated when SLX-4^Δ379–402^ was artificially tethered to another CO designation factor ZHP-3, using the same nanobody method, demonstrating that enforced spatial localization of SLX-4 at the CO designation site is sufficient to trigger CO formation. Notably, although artificially recruited SLX-4^Δ379–402^ forms bright foci at designated CO sites, endogenous SLX-4 in wild type exhibits diffuse nuclear distribution during late pachytene. Although we observe small aggregates of wild-type SLX-4, these structures show no colocalization with CO markers such as COSA-1 at CO designation sites. Although the function of these aggregates remains unclear, our data suggest that HJ resolution at CO sites may require only trace amounts of SLX-4. Moreover, this catalytic process could be highly dynamic and SLX-4 may execute cleavage without prolonged residence at CO sites. Given that both COSA-1 and ZHP-3 are expressed exclusively during meiotic prophase I, the artificially enforced SLX-4 tethering should be transient. This temporal restriction ensures that functions of SLX-4 in other cell cycle stages, such as somatic DNA repair, are intact.

As a scaffold, SLX-4 can assemble three structure-specific endonucleases into a trinuclease complex (*31*). The interaction between SLX-4 and these endonucleases is highly regulated, and our finding that COSA-1 binding increases SLX-4–endonuclease interaction supports this. Artificially driving SLX-4–MUS-81 complex formation in S phase triggers DNA damage toxicity (*32*–*34*). An outstanding question raised by our work is the precise role of COSA-1 in this process: whether it functions primarily as a scaffold for SLX-4 recruitment, as a cyclin activating CDK-2 kinase, or both. Our data favor a model where COSA-1 likely serves these dual roles in a coordinated manner. The COSA-1^R163A^ mutation that disrupts SLX-4 binding does not affect its interaction with CDK-2, indicating that COSA-1 has distinct interfaces for these partners. This allows it to act as a scaffold recruiting SLX-4 to designated CO sites while simultaneously functioning as the cyclin partner for CDK-2.

Furthermore, these two roles may be mechanistically linked through phosphorylation. A previous study showed that phosphorylation of SLX-4 by CDK-1–cyclin B drives the folding of a canonical SAP domain in the MUS-81 binding region and facilitates the interaction with MUS-81–EME1 in early mitosis (*35*). An analogous regulatory mechanism may exist during *C. elegans* meiosis*.* First, *C. elegans* SLX-4 contains several CDK consensus motifs (S/TP), one of which (T471; T1561 in human SLX-4) is a conserved phosphorylation site and critical for binding to MUS-81. Second, COSA-1–CDK-2 is active during meiotic prophase and is known to phosphorylate CO designation factors such as MSH-5 and ZHP-3/4 at multiple sites in the C-terminal tail and contribute to the pro-CO activity of the MutSγ complex and ZHP-3/4 (*36*, *37*). It is plausible that COSA-1–CDK-2 not only facilitates the localization of SLX-4 but also phosphorylates it to promote the recruitment or activation of endonucleases for efficient HJ resolution. Therefore, future studies are needed to explore the potential phosphorylation of SLX-4 by COSA-1–CDK-2 and its functional consequences. All in all, our study links CO designation with HJ resolution directly, and we provide evidence that the mechanism may be fundamentally conserved.

## MATERIALS AND METHODS

### *C. elegans* strains and CRISPR-Cas9–based genome editing

All *C. elegans* strains used in this study were maintained on nematode growth medium (NGM) plates seeded with *Escherichia coli* OP50 (a uracil-auxotrophic strain) and cultured at 20°C. Most genome editing was performed in the N2 Bristol (wild-type) background. All CRISPR-Cas9–edited strains were back-crossed to N2 for four generations before analysis. Sequences of all the oligos and CRISPR RNAs (crRNAs) are provided in table S2. Strains used in this study are listed in table S3.

To generate insertion, deletion, or point mutation *C. elegans* strains, young adult N2 hermaphrodites were microinjected with a mixture (10 μl) comprising 0.5 μl of Cas9 (4 mg/ml; GenScript, #Z03702S) complexed with 1 to 2 μg of a single sgRNA, 50 ng of the coinjection marker plasmid pCFJ90, and a repair template consisting of 300 to 600 ng of melting double-stranded DNA (dsDNA) donor cocktail or 1 to 1.5 μg of ssDNA templates (*38*).

For the COSA-1 (R163A) edit, we first generated the *cosa-1::mBaojin* knockin strain. The mBaojin sequence was amplified from the pSF11-mBaojin plasmid (vector ID: 0000449) from WeKwikGene (https://wekwikgene.wllsb.edu.cn/), Westlake Laboratory of Life Sciences and Biomedicine (*25*). On the basis of this strain, the *cosa-1^R163A^::mBaojin* point mutation was subsequently introduced using an ssDNA template. Nucleotides 454 to 459 of the COSA-1 cDNA (wild-type CTTGAA) were silently mutated to CT**c**GA**g** (bolded letters indicate the specific nucleotides that were altered in the silent mutation). For genotyping, an ~300–base pair (bp) fragment encompassing both the engineered Xho I site and the R163A mutation was amplified from single-worm lysates and digested with Xho I.

### TurboID-based enzymatic protein labeling and extraction of biotinylated proteins from *C. elegans*

Nuclei extracted from *C. elegans* were used for the analysis. Gravid adults were bleached and the eggs were transferred to NGM plates seeded with OP50 to obtain 15 ml of synchronous adult worms (N2; *cosa-1::3×HA::TurboID*). Worms were washed off the plates with M9 into a conical flask, 1 mM biotin (Sangon, #A600078) was added, and the suspension was incubated at 25°C with 60 rpm for 2 hours. The worms were washed three times in M9 buffer and left on ice to settle after incubation. The worms were mixed with small shards of broken glass coverslips and shredded by vortexing in 15-ml conical tubes containing ice-cold nuclear isolation buffer [10 mM Hepes (pH 7.6), 1 mM EGTA, 1.5 mM MgCl_2_, 10 mM KCl, 250 mM sucrose, 0.5 mM dithiothreitol (DTT), and protease inhibitor cocktail]. Glass fragments were removed by centrifugation at 30*g* for 1 min, and worm debris was eliminated at 350*g* for 1 min. The supernatant containing nuclei was first passed through a 100-μm mesh and then filtered again through a 40-μm mesh. Last, nuclei were collected by centrifugation at 2500*g* for 10 min. SDS and DTT were added to the sample to final concentrations of 1% and 10 mM, respectively, and the mixture was immediately heated at 95°C for 10 min, followed by sonication (15% continuous output; SCIENTZ JY92-IIN). The sample was then adjusted to 2 M urea with a stock solution [8 M urea, 1% SDS, 50 mM tris-HCl (pH 7.4), and 150 mM NaCl] and centrifuged at 14,000 rpm for 40 min. The clear supernatant between the solid and the surface lipid layer was transferred to a new tube and loaded twice onto Zeba spin desalting columns (7K MWCO, Thermo Fisher Scientific) to remove free biotin. Last, Dynabeads MyOne Streptavidin T1 (Invitrogen, 600 μl/1.5 ml of nuclei) were added, and the mixture was incubated overnight at room temperature to capture biotinylated proteins. The magnetic beads were washed as follows: two times with 150 mM NaCl, 1 mM EDTA, 2% SDS, and 50 mM tris-HCl (pH 7.4); once with 1× tris-buffered saline (TBS); two times with 1 M KCl, 1 mM EDTA, 50 mM tris-HCl, and 0.1% Tween 20 (pH 7.4); two times with 0.1 M Na_2_CO_3_ and 0.1% Tween 20 (pH 11.5); and two times with 2 M urea, 10 mM tris-HCl, and 0.1% Tween 20 (pH 8.0). Last, the beads were washed five times with 1× TBS. For Western blot sample preparation, 20-μl beads were boiled in SDS sample buffer with 80 mM biotin (Sangon, #A600078) for 15 min. The remaining beads were stored at −80°C.

### On-bead protein digestion

Bead samples were taken out from −80°C and thawed on ice. The samples were placed on a magnetic stand for 2 min to collect the beads. The beads were then resuspended in 80 μl of lysis buffer [2 M urea, 50 mM Hepes, 1 mM DTT, and 0.4 μg of trypsin (pH 8.0)] and incubated at 37°C with shaking at 1000 rpm for 60 min. After incubation, the samples were placed on a magnetic stand for 2 min and the supernatant was transferred to a new centrifuge tube. The beads were washed with 60 μl of lysis buffer [2 M urea, 50 mM Hepes, and 1 mM DTT (pH 8.0)] and incubated at 37°C with shaking at 1000 rpm for 5 min, following another 2-min magnetic separation, and the supernatant was combined with the previous fraction. The pooled supernatant was reduced with 4 mM DTT at 37°C for 30 min and alkylated with 10 mM iodoacetamide (IAM) at room temperature in the dark for 45 min. Digestion was performed with 0.5 μg of trypsin at 37°C overnight. Peptides were acidified with 10% formic acid (FA) to reach a final concentration of 1% and then desalted using the SP2 method (*39*). Samples were dried in a SpeedVac and reconstituted in 0.1% FA for mass spectrometry (MS) analysis.

### LC-MS/MS analysis

All nanoflow liquid chromatography–tandem mass spectrometry (nanoLC-MS/MS) experiments were performed on an Orbitrap Exploris 480 (Thermo Fisher Scientific) equipped with a Vanquish Neo UHPLC system (Thermo Fisher Scientific). Peptides were loaded on a trap column [75-μm inside diameter (ID) by 2 cm; Acclaim PepMap 100, Thermo Fisher Scientific) and then separated onto a C18 column packed with reversed-phase silica (75-μm ID by 20 cm; Reprosil-Pur 120 C18-AQ, 1.9 μm, Dr. Maisch GmbH). The peptides bound on the column were eluted with a 120-min linear gradient. The solvent A consisted of 0.1% FA in water and solvent B consisted of 80% acetonitrile and 0.1% FA. The segmented gradient was as follows: 6% B, 1.8 min; 6 to 6.5% B, 0.2 min; 6.5 to 28% B, 83 min; 28 to 38% B, 21 min; 38 to 90% B, 4 min; 90 to 99% B, 1 min; and 99% B, 9 min at a constant flow rate of 400 nl/min. With the data-independent acquisition mode, the MS scans were acquired at a high resolution of 120,000 [mass/charge ratio (*m/z*) 200] across the mass range of 400 to 1100 *m/z*. The AGC (Automatic Gain Control) target value was 3 × 10^6^ with a maximum injection time of 22 ms. The MS/MS scans were acquired with 40 variable overlapping precursor isolation windows and centered at 410, 427.5, 442.5, 455.5, 467, 478, 488.5, 498, 507.5, 517.5, 527.5, 537, 546.5, 556.5, 566.5, 576, 585.5, 595.5, 606.5, 617.5, 627.5, 638.5, 650, 661, 672, 683.5, 696, 709.5, 724, 740, 757.5, 775.5, 794, 813.5, 837.5, 868.5, 907, 953.5, 1009, and 1089.5. Programmed normalized collision energy was set to 25, 27, and 30%. The AGC target value was 1 × 10^6^ with a maximum injection time of 40 ms in MS2. For the nanoelectrospray ion source setting, the spray voltage was 2.1 kV, there is no sheath gas flow, and the heated capillary temperature was 320°C.

### DIA data processing

The DIA (Data-Independent Acquisition) proteomics data were analyzed using DIA-NN (version 1.8.1) in library-free mode (*40*). Protein sequences for database search were reviewed from the *C. elegans* proteome (UP000001940_6239.fasta, 19,832 entries, download date: 3 July 2024). Trypsin (full) was specified as the proteolytic enzyme allowing up to two missed cleavages. The mass tolerance for both precursor and product ions were set to default. Carbamidomethylation of Cys was set as fixed; oxidation of Met and N-terminal acetylation was set as variable. The range of peptide length was set from 7 to 50. The run-specific false-discovery rate (FDR) of identifications at both peptide and protein levels was estimated by Percolator (*41*). Precursor and protein FDRs were set to 1%. All the other parameters in DIA-NN were set to default values.

### Yeast two-hybrid analysis

Yeast two-hybrid assays were performed as previously described (*42*). Briefly, the full-length coding sequences (or mutant variants) of the two proteins to be tested were cloned into pGADT7 (GAL4 AD fusion) and pGBKT7 (GAL4 DNA binding fusion), respectively, and cotransformed into yeast strain AH109. Successful cotransformants were first selected on medium lacking tryptophan (-Trp) and leucine (-Leu), and positive interactors were subsequently identified on medium additionally depleted of histidine (-His).

### Yeast three-hybrid analysis

All transformations were performed with the AH109-pBridge Yeast Three-Hybrid Media Kit (Coolaber, #YM3000-1Set). The transformation protocol was identical to that of the conventional yeast two-hybrid assays, but cotransformation selection was carried out on SD/-Leu/-Met/-Trp plates, and the enhancement of SLX-4–nuclease interactions by COSA-1 was assessed on SD/-His/-Leu/-Met/-Trp medium. Plates were kept at 25°C for 3 to 5 days.

### Immunofluorescence

Young adults (24 hours post-L4) were dissected in 6 μl of 1× Egg Buffer [25 mM Hepes (pH 7.3), 118 mM NaCl, 48 mM KCl, 2 mM CaCl_2_, 2 mM MgCl_2_, and 0.1% Tween 20] and briefly fixed in 1% formaldehyde (Sigma-Aldrich, #MKCJ8456). The body was trimmed away, and the gonads were flash frozen in liquid nitrogen, freeze cracked, and put in −20°C methanol for 10 min. Following three 10-min washes in 1× PBST (phosphate-buffered saline with 0.1% Tween 20), gonads were blocked with 1% bovine serum albumin (BSA) in PBST for 30 min at room temperature. The primary antibody was incubated overnight at 4°C at the indicated dilutions: mouse anti-FLAG (1:800; Sigma-Aldrich, #F1804), mouse anti-HA (1:500; BioLegend, #16B12), GFP-Booster (1:400; ChromoTek, #gb2AF488), rabbit anti-HIM-3 (1:300; generated by ABclonal Technology), rabbit anti-RAD-51 (1:1000; generated by ABclonal Technology), and rabbit anti-phospho-H3 (Ser^10^; 1:100; Fisher Scientific). Following three 10-min PBST washes, samples were incubated with secondary antibodies (1:750; Invitrogen, Alexa Fluor 488 or Alexa Fluor 594) and DAPI (1:20; Sangon Biotech, #E607303) for 2 hours at room temperature. For *cosa-1::3×HA::TurboID*, samples were incubated with streptavidin–Alexa Fluor 488 (1:500; Fisher Scientific, S11223) at room temperature for 2 hours after 1% BSA blocking. After three 15-min PBST washes, samples were mounted in Vectashield H-1000 antifade medium and sealed with clear nail polish. Images were acquired using an Olympus SpinSR10 microscope [60×/1.42–numerical aperture (NA) oil objective], and *Z*-stacks of optical sections spanning 5 to 10 μm were acquired at a 0.5-μm *z*-spacing. Ultrahigh-resolution imaging was performed on a ZEISS Elyra 7 SIM system equipped with a 63×/1.40-NA oil objective at a 0.25-μm *z*-spacing.

### Analyses of brood size, progeny viability, and incidence of male

To quantify brood size, progeny viability, and male frequency, four biological replicates were set up for each genotype. Three L4 hermaphrodites were placed on a single counting plate per replicate and transferred to fresh plates every 12 hours; eggs laid after each transfer were counted. Egg-laying ceased after ~5 days, and the cumulative brood size was recorded. Unhatched eggs were counted 24 hours later, and the percentage of progeny viability was calculated as the total number of viable progenies divided by the total number of eggs. When F1 progeny reached the L4/adult stage, males were counted. The incidence of males was calculated as the number of males divided by the total number of viable progenies.

### Immunoblots

The prepared protein samples were separated using Super-PAGE (Epizyme, #LK403 and #LK409) and transferred onto polyvinylidene difluoride (PVDF) membranes (Millipore, IPVH00010). PVDF membranes were blocked with 5% nonfat milk in TBST for 1 hour at room temperature and then incubated with primary antibodies overnight at 4°C with gentle agitation (20 rpm). Antibodies were diluted as follows: mouse anti-FLAG (1:1000; Sigma-Aldrich, #F1804), mouse anti-HA (1:1000; BioLegend, #16B12), rabbit anti-β-Actin (1:100,000; ABclonal Technology, #AC026), and rabbit anti-HIM-3 (1:1000; generated by ABclonal Technology).

### Protein depletion by the auxin-inducible degradation system

Auxin-inducible degradation (AID) of SLX-4 from the *C. elegans* germ line was performed as previously described (*43*, *44*). Briefly, NGM plates containing 3 mM 1-naphthaleneacetic acid potassium salt (K-NAA; a synthetic auxin analog) were seeded with 400 μl of OP50 also containing 3 mM K-NAA. L4 larvae were fed on these plates at 20°C for 12 or 24 hours before cytological analysis.

### Statistics

All statistical analyses and graphs were generated using GraphPad Prism 8 software. Differences in brood size, progeny viability, incidence of males, the number of DAPI-stained bodies in diakinesis, and the number of ZHP-3 foci were evaluated by the *t* test. *P* < 0.05 was considered significant.
