# Supplementary material for: COSA-1–SLX-4 interaction directly links crossover designation with Holliday junction resolution
Source: Sci Adv. 2026 Apr 24;12(17):eadx9148. doi: 10.1126/sciadv.adx9148 (PMC13108560; doi:10.1126/sciadv.adx9148)
Supplement: Supplementary file 1 — Figs. S1 to S11 Tables S1 to S3 [file sciadv.adx9148_sm.pdf]

Science Advances
AAAS

Supplementary Materials for

COSA-1–SLX-4 interaction directly links crossover designation with Holliday junction resolution

Guoteng Liu *et al.*

Corresponding author: Hongtao Zhang, ht.zhang@sdu.edu.cn; Ye Hong, hongye@sdu.edu.cn

Sci. Adv. **12**, eadx9148 (2026)
DOI: 10.1126/sciadv.adx9148

This PDF file includes:

Figs. S1 to S11
Tables S1 to S3

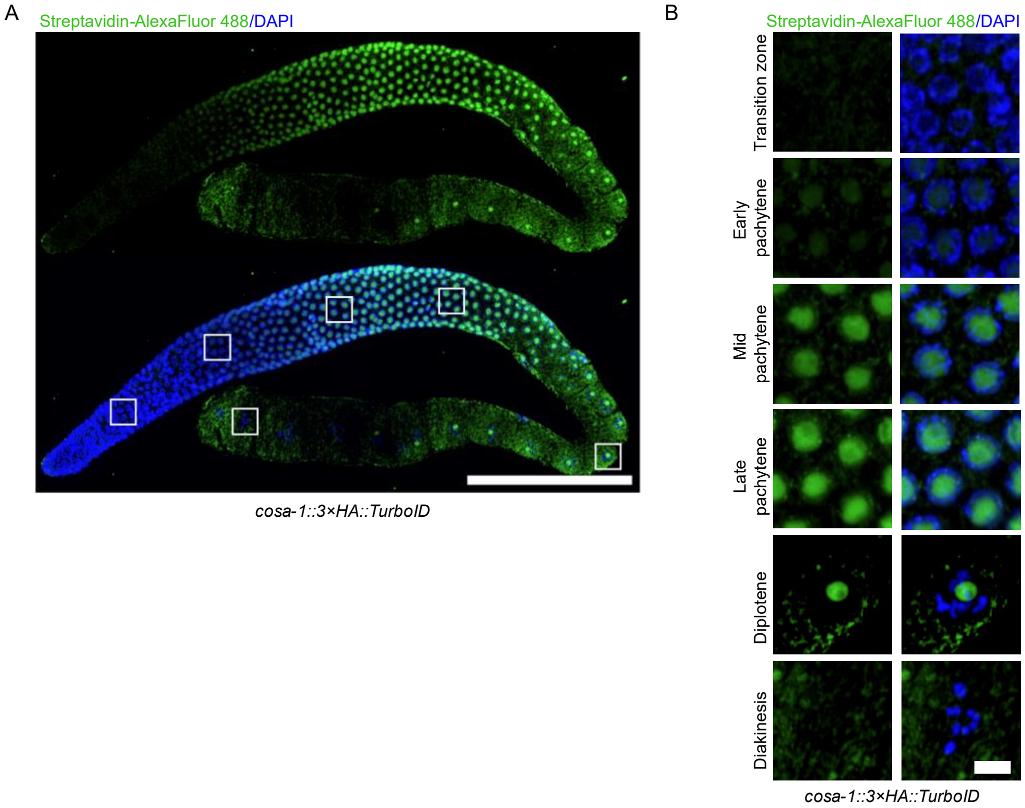

Figure S1 Biotinylation signal in the gonad of the *cosa-1::3×HA::TurboID* strain

(A) Gonad from a *cosa-1::3×HA::TurboID* worm stained with DAPI (blue, DNA) and Streptavidin-AlexaFluor 488 (green, biotinylated proteins). Scale bar, 100 µm. (B) Representative images of nuclei at the indicated meiotic prophase stages. Scale bar, 5 µm.

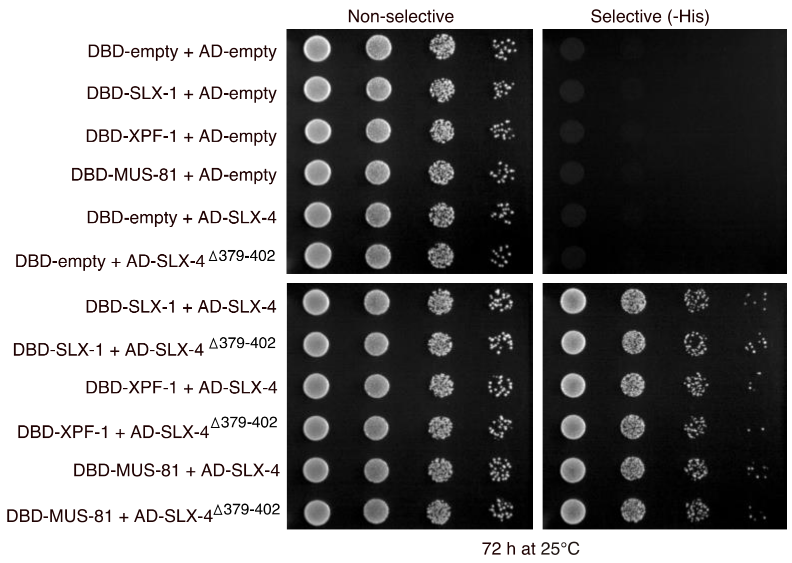

Figure S2 Examination of the interaction between SLX-4, SLX-4$^{\Delta 379–402}$, XPF-1, SLX-1 and MUS-81 by the yeast two-hybrid assay

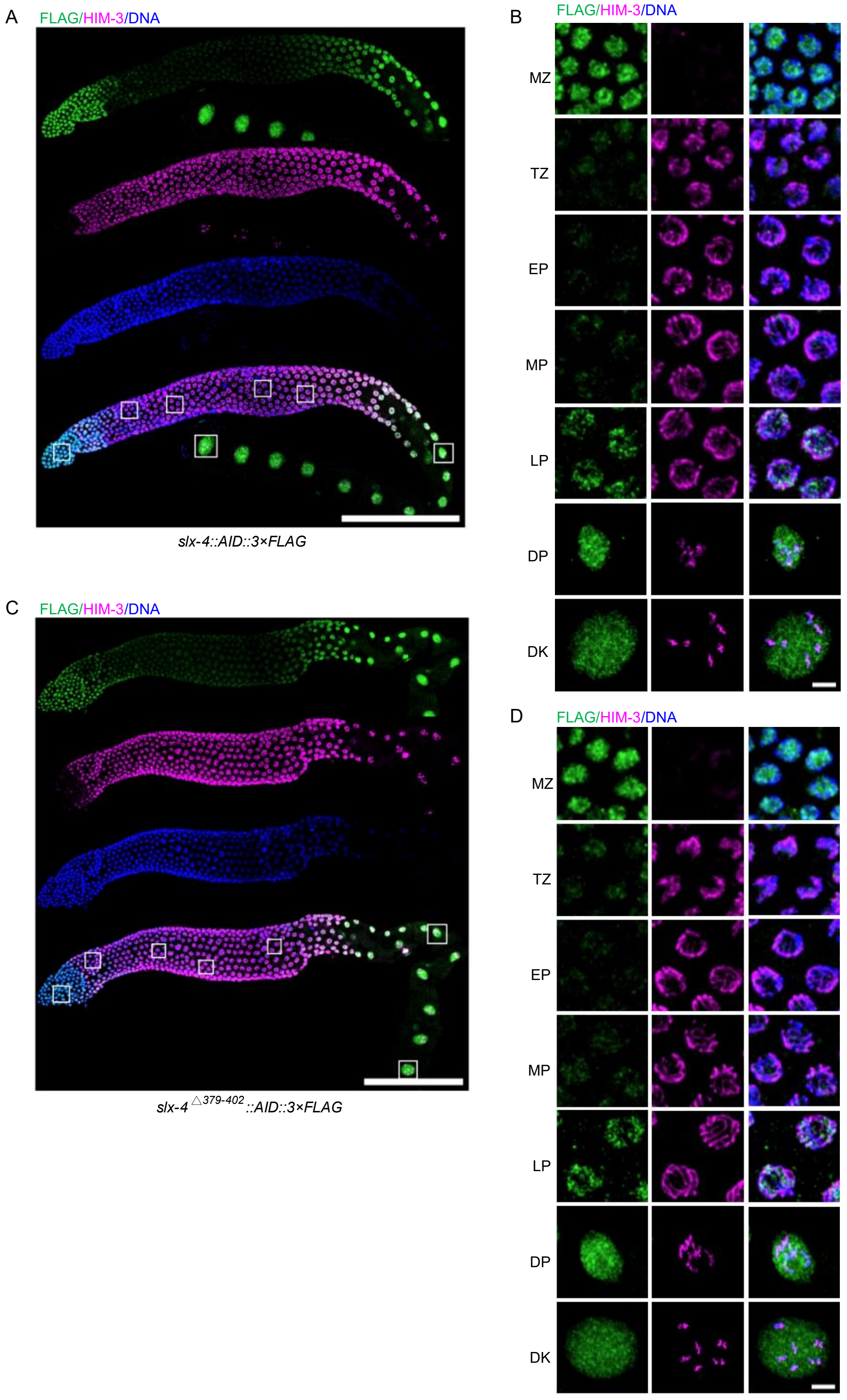

Figure S3 Analysis of the localization of SLX-4 and SLX-4$^{Δ379–402}$ in germline nuclei

(A) Localization of SLX-4 in germline nuclei. Scale bar, 100 μm. (B) Representative nuclear images across meiotic stages revealing SLX-4 expression dynamics and

subcellular localization. MZ: Mitotic zone, TZ: Transition zone, EP: Early pachytene, MP: Mid pachytene, LP: Late pachytene, DP: diplotene, DK: diakinesis. Scale bar, 5 µm. (C) Immunolocalization of SLX-4$^{\Delta 379–402}$ in germline nuclei. Scale bar, 100 µm. (D) Representative images of nuclei of different meiotic stage showing the expression and localization of SLX-4$^{\Delta 379–402}$. Scale bar, 5 µm.

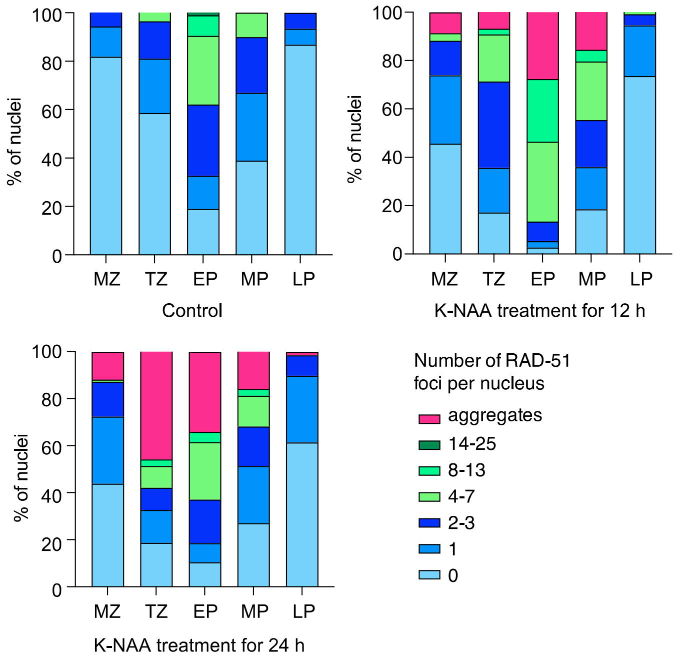

Figure S4 Analysis of RAD-51 foci/aggregates after SLX-4 depletion Auxin-inducible degradation of SLX-4 (12 or 24 h K-NAA treatment) in *slx-4::AID::3×FLAG; TIR1* worms led to increased RAD-51 foci/aggregates. TZ: Transition zone, MZ: Mitotic zone, EP: Early pachytene, MP: Mid pachytene, LP: Late pachytene.

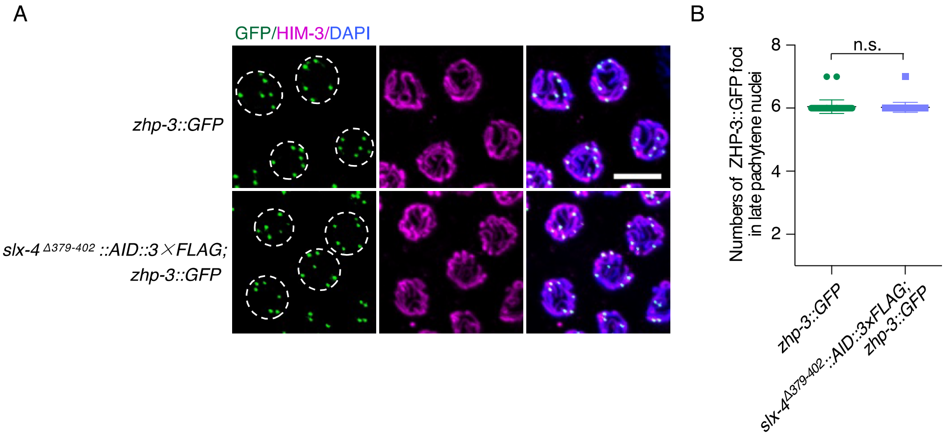

Figure S5 Localization of ZHP-3::GFP in late pachytene nuclei of *slx-4*$^{\Delta 379–402}$*::AID::3×FLAG*; *zhp-3::GFP* worms

(A) Representative images of late pachytene nuclei of the indicated genotypes stained with GFP booster to detect ZHP-3 (green), antibodies against HIM-3 (magenta), and counterstained with DAPI (blue). Scale bar, 5 µm. (B) Quantification of ZHP-3::GFP foci in the late pachytene nuclei of *zhp-3::GFP* and *slx-4*$^{\Delta 379–402}$*::AID::3×FLAG*; *zhp-3::GFP* mutants.

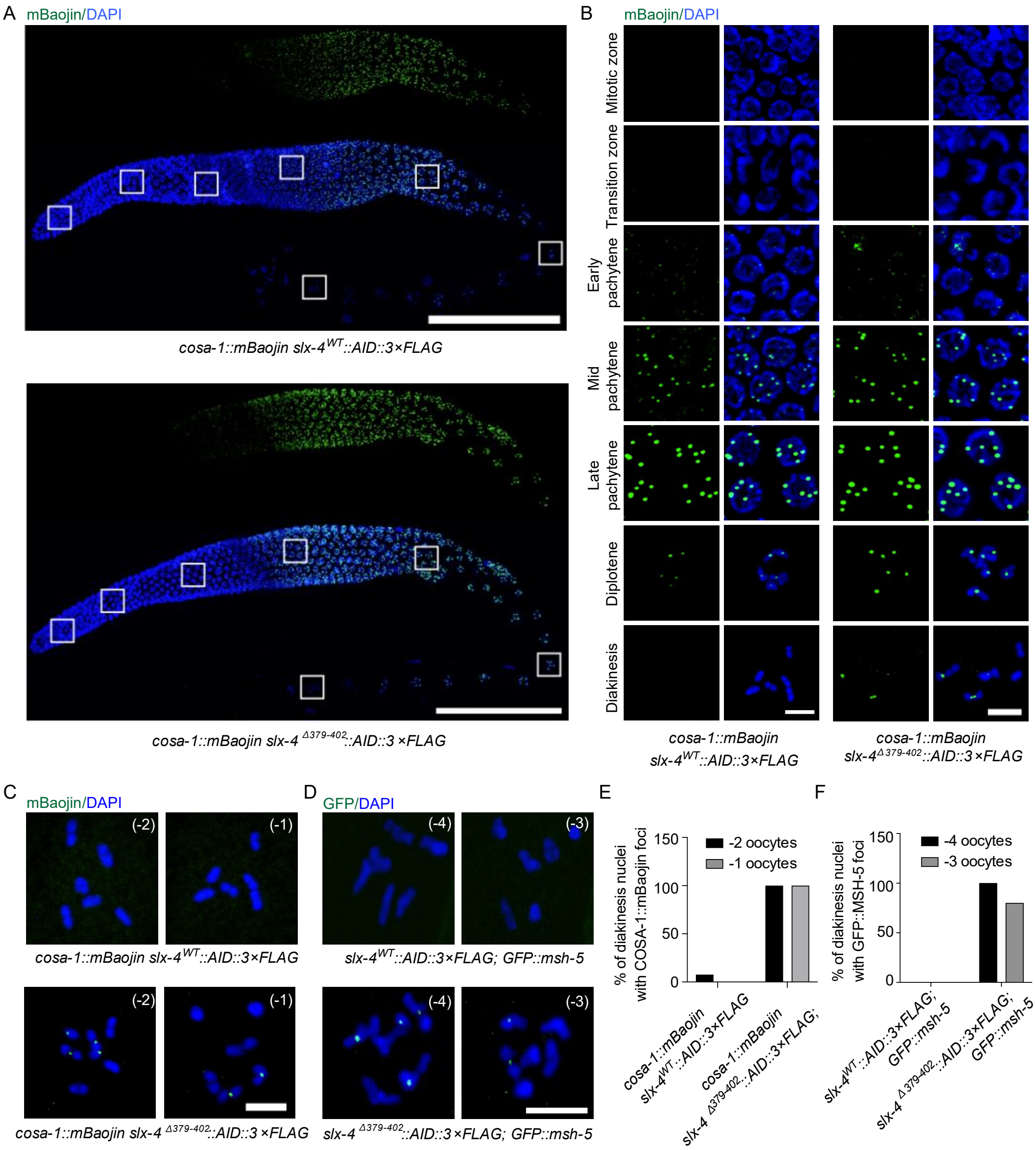

Figure S6 Persistent localization of MSH-5 and COSA-1 on diakinesis chromosomes in *slx-4*$^{\Delta 379\text{–}402}$ mutants (A) Germline images of the *cosa-1::mBaojin slx-4*^{WT}*::AID::3×FLAG* and *cosa-1::mBaojin slx-4*$^{\Delta 379\text{–}402}$*::AID::3×FLAG*. Scale bar, 100 μm. (B) Representative images of nuclei at indicated meiotic prophase stages, showing COSA-1 (green) and DAPI (blue). Scale bar, 5 μm. (C, D) Representative diakinesis nuclei showing persistent foci of (C) COSA-1::mBaojin or (D) GFP::MSH-5 (green), with DAPI (blue). Scale bar, 5 μm. (E, F) Quantification of the percentage of diakinesis nuclei with COSA-1::mBaojin foci (E) or GFP::MSH-5 foci (F).

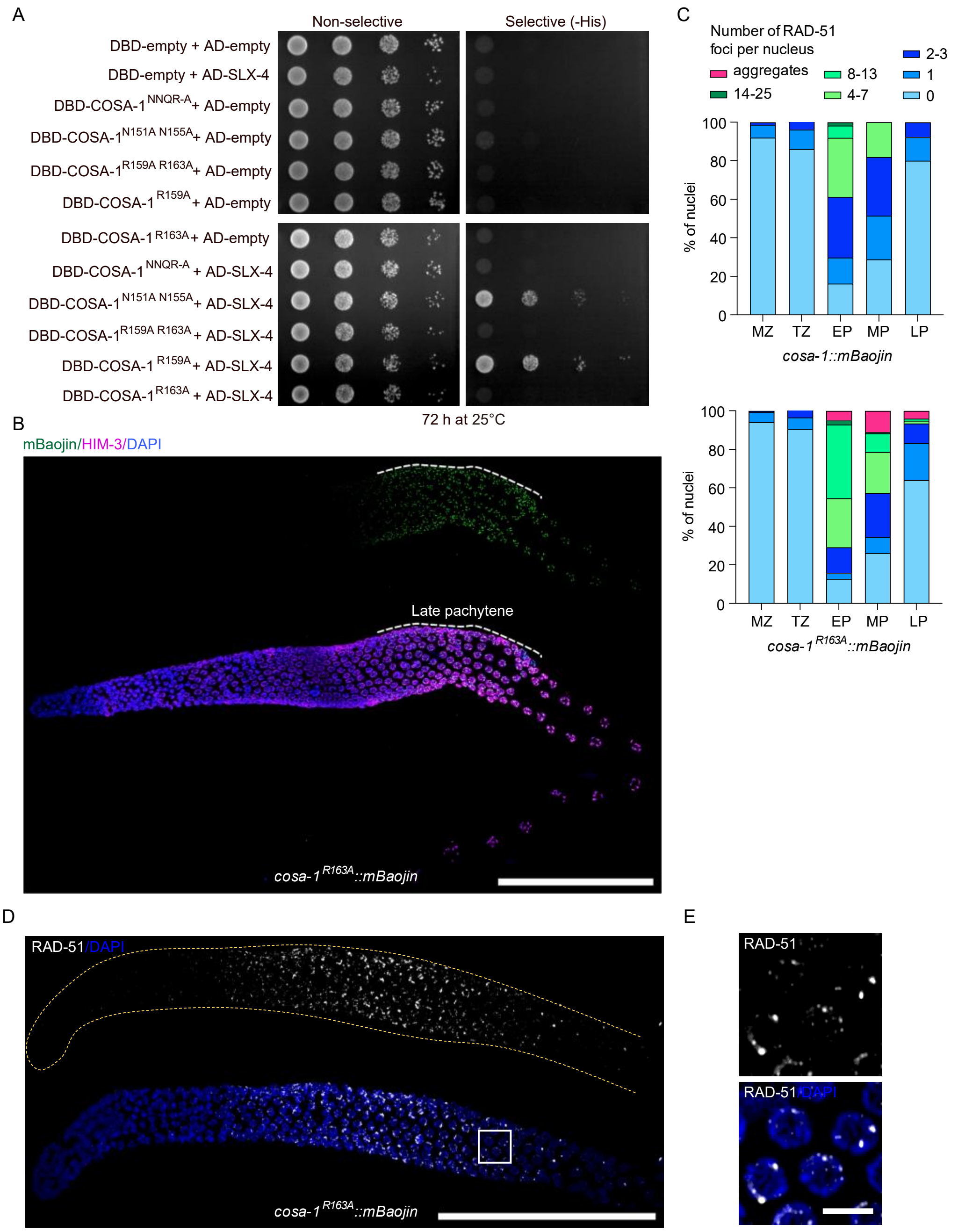

Figure S7 Analysis of the COSA-1 R163A mutation that disrupts SLX-4 interaction (A) Analysis of the amino acids on COSA-1 involved in the interaction with SLX-4 by the yeast two-hybrid assay. NNQR-A means that the Asn151, Asn155, Gln159 and Arg163 were mutated to Alanine. (B) Whole-gonad imaging showing HIM-3 (magenta), DAPI (blue) and *COSA-1^{R163A}::mBaojin* (green). Scale bar, 100 µm. (C) Quantification of RAD-51 foci in indicated regions of the germline. MZ: Mitotic zone, TZ: Transition zone, EP: Early pachytene, MP: Mid pachytene, LP: Late pachytene. (D) Germline image of the *cosa-1^{R163A}::mBaojin* mutant stained for RAD-51 (white) and

DAPI (blue). Scale bar, 100 μm. (E) Representative nuclei from the *cosa-1*^{R163A}*::mBaojin* mutant showing RAD-51 foci or aggregates. Scale bar, 5 μm.

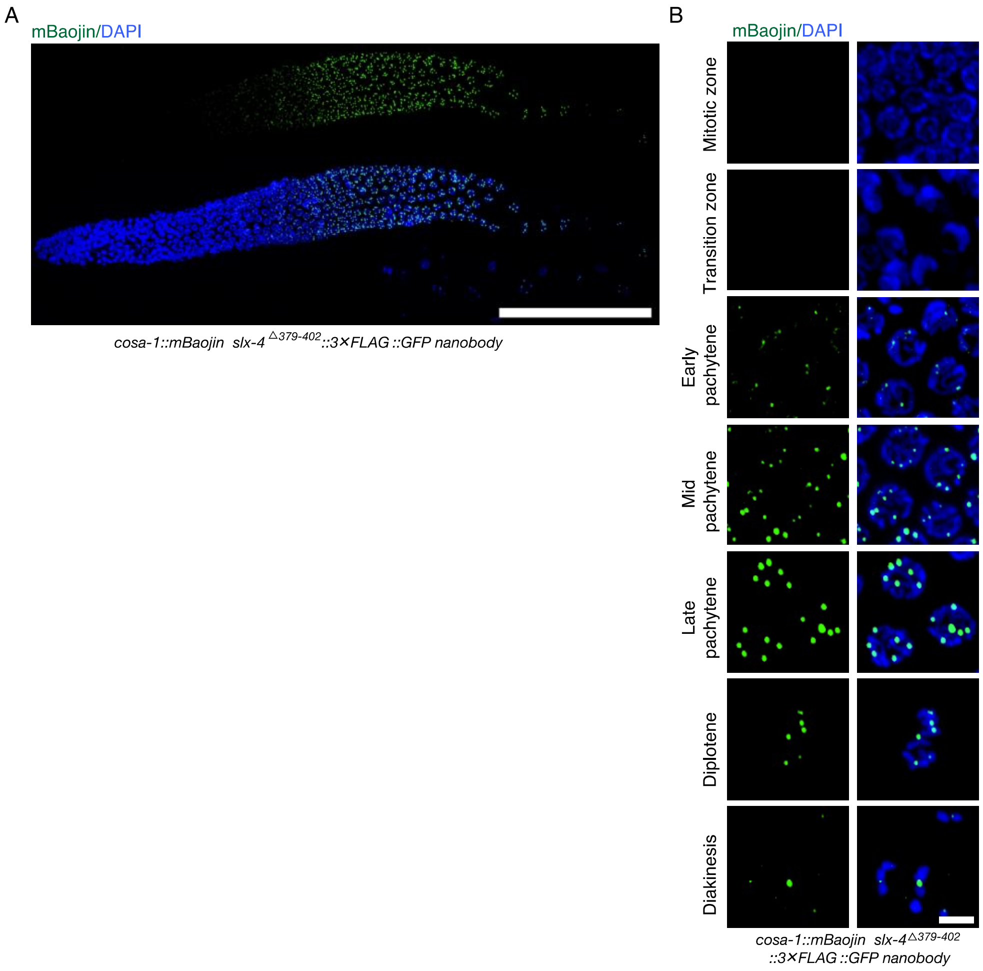

Figure S8 Localization of COSA-1 in *slx-4*$^{\Delta 379\text{-}402}$*::3×FLAG::GFP nanobody* mutant (A) Germline of the *cosa-1::mBaojin slx-4*$^{\Delta 379\text{-}402}$*::3×FLAG::GFP nanobody* mutant. Scale bar, 100 µm. (B) Representative images of nuclei at indicated meiotic prophase stages, showing COSA-1 (green) and DAPI (blue). Scale bar, 5 µm.

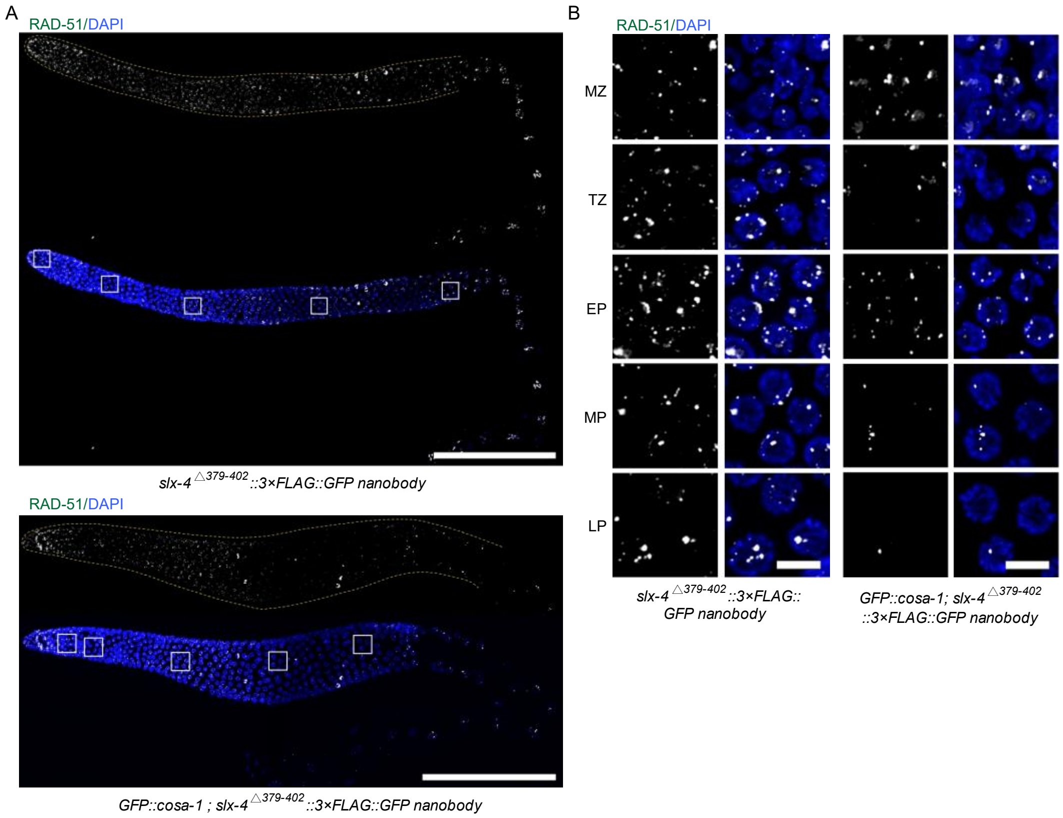

Figure S9 Artificially tethering SLX-4$^{\Delta 379\text{-}402}$ to the CO designation sites rescued the aberrant accumulation of RAD-51 foci in meiotic prophase (A) Images of germline stained with RAD-51 antibody for the indicated genotypes. Scale bar, 100 μm. (B) Representative images of RAD-51 staining in different stages of meiotic prophase for the indicated genotypes. MZ: Mitotic zone, TZ: Transition zone, EP: Early pachytene, MP: Mid pachytene, LP: Late pachytene. Scale bar, 5 μm.

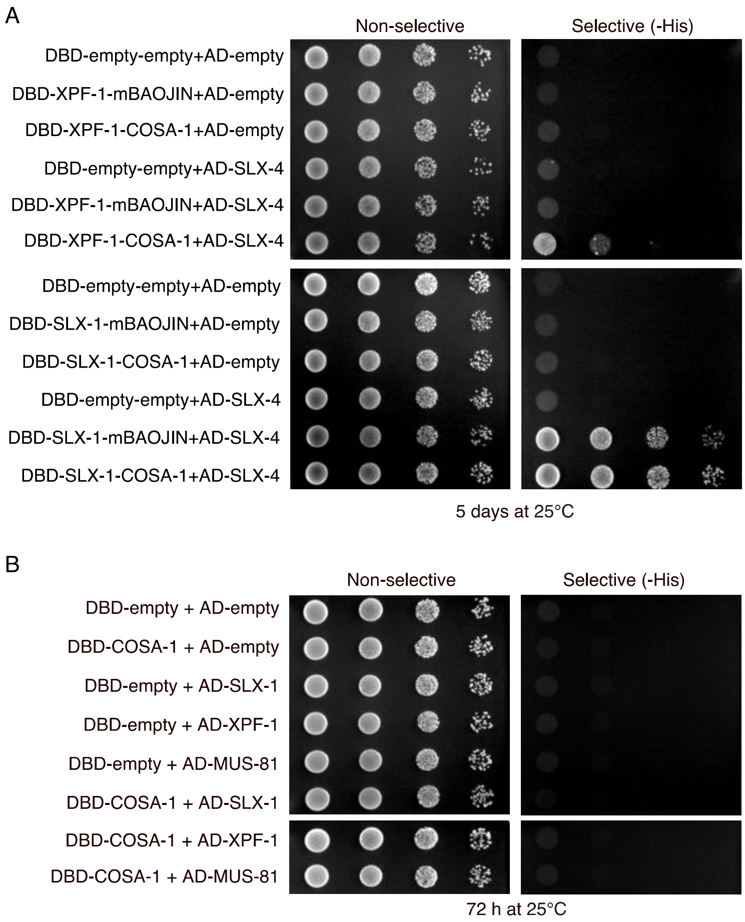

Figure S10

(A) Analysis of COSA-1-mediated modulation of SLX-4-XPF-1 and SLX-4-SLX-1 interactions by yeast three-hybrid assays. (B) Examination of the interaction between COSA-1, XPF-1, SLX-1 and MUS-81 by yeast two-hybrid assays.

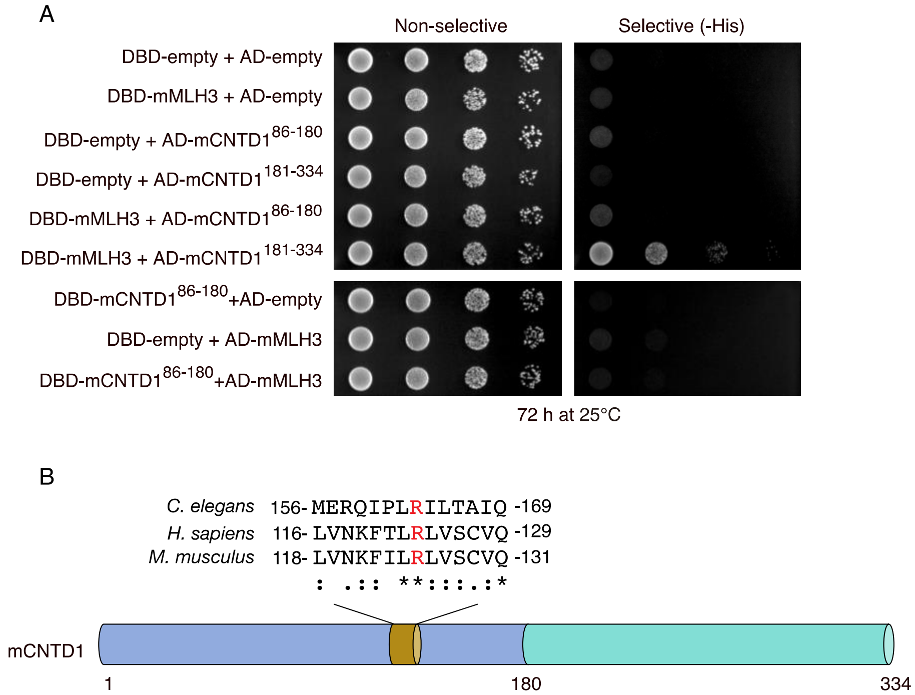

Figure S11 Mapping the MLH3-binding region on mCNTD1 (A) Analysis of mMLH3 interaction region by yeast two-hybrid assay. (B) Schematic illustration of mCNTD1. The MLH3 interaction region is in green. The conserved arginine involved in SLX-4 interaction in *C. elegans* are highlighted in red.

Table S1 Quantification of brood size, progeny viability, and male frequency for strains generated in this study

Genotype	Average brood size (n)[a]	% Progeny viability[b]	% Males[c]
cosa-1::mBaojin	308.67 (12)	99.77	0.19
cosa-1^{R163A}*::mBaojin*	310.08 (12)	35.66	12.07
slx-4::AID::3×FLAG	306.67 (12)	99.33	0.13
slx-4$^{\triangle 379\text{-}402}$*::AID::3×FLAG*	225.75 (12)	49.43	10.63
slx-4::3×FLAG::GFP nanobody	290.33 (12)	99.00	0.2
slx-4$^{\triangle 379\text{-}402}$*::3×FLAG::GFP nanobody*	164.58 (12)	35.21	10.31
spo-11	191.00 (12)	ND	ND
cosa-1^{R163A}*::3×HA::TurboID*	307.58 (12)	7.51	ND

[a]individual hermaphrodites; complete brood sizes scored, n = number of worms analyzed,
[b]fertilized eggs scored,
[c]adults scored,
ND, not determined.

Table S2 crRNAs, repair templates and genotyping primers /restriction enzyme for mutant alleles generated in this study

Allele	crRNAs and long/short repair templates & single-stranded templates	Genotyping primers/restriction enzyme and fragment sizes
cosa-1::mBaojin	5’-cagagatggtagTTACGAGG-3’<br>5’-TCTCGGCACTTCTCAAATTGCCAATGTTT<br>CAGTGATAATCAATAATTTATTGAGATGTGA<br>CAATGCTTATGTCGAACCATTAAAGCAATCG<br>ATTATAGAGCTCGCCTGCGCGAAAAAGgtaact<br>gctggccgagtttttctaggccacgcgtggcaattttacaattaattatttt<br>ttatttatttcagAATGAGAGTATTCCGGAATGCAGC<br>ACCTCCTCGggaggtggcggatctggaggtggaggctctgga<br>ggaggtggatctATGGTATCCAAGGGAGAAGAAG<br>AAAACATGGCTAGTACCCCATTCAAGTTCCA<br>GCTTAAGGGAACTATTAACGGAAAGTCTTTC<br>ACTGTTGAAGGAGAAGGAGAAGGAAACTC<br>TCACGAAGGATCTCATAAGGGAAAGTACGT<br>TTGCACTTCTGGAAAGCTTCCAATGTCTTGG<br>GCTGCTCTTGGAACTACTTTCGGATATGGAA<br>TGAAGTATTACACTAAGTACCCATCTGGACT<br>TAAGgtaagtttaaacatatatatactaactaaccctgattatttaaattt<br>tcagAACTGGTTCCGCGAAGTTATGCCAGGAG<br>GATTCACTTATGATCGCCATATCCAGTACAA<br>GGGAGATGGATCTATCCATGCTAAGCATCAG<br>CATTTCATGAAGAATGGAACTTACCACAACA<br>TCGTTGAATTCACTGGACAAGATTTCAAGgta<br>agtttaaacagttcggtactaactaaccatacatatttaaattttcagGA<br>AAACTCCCCAGTTCTTACCGGAGATATGAAC<br>GTTTCCCTTCCAAACGAAGTTCCACAAATCC<br>CACGTGATGATGGAGTTGAGTGCCCAGTTA<br>CCCTTCTTTACCCACTTCTTTCCGATAAGTCC<br>AAGTACGTTGAGGCTCATCAGTACACTATCT<br>GCAAGCCACTTCATAACCAACCAGCTCCAG<br>ATGTTCCATACCATTGGATCCGCAAGCAATA<br>CACTCAATCTAAGGATGATGCTGAGGAGCG<br>CGATCACATCTGCCAATCTGAGACTCTTGAG<br>GCTCACCTTAAGGGAATGGATGAGCTTTACA<br>AGTAATAActaccatctctgacagcacctctttgtcgccgattcca<br>ctggtcgcggctcgttcactgcaacaaattattgatttttattgtcatgtacc<br>atattgaatgcataatgtttaatttaataaatttggatttagttttataaatatgt<br>ttcatttttcaatgaaatcatccaaaaattcaaatttgcgcgtaaagtatgg<br>agttttcgcaatttcctg-3’	F 5’-GTGACAATGCTTATGTCGAACC<br>AT-3’<br>R 5’-gtggtgcaatgagtacgtgac-3’<br>WT: 407 bp<br>Mutant: 1259 bp

	5’-TGCAGCACCTCCTCGggaggtggcggatctggagg	
	tggaggctctggaggaggtggatctATGGTATCCAAGGG	
	AGAAGAAGAAAACATGGCTAGTACCCCATT	
	CAAGTTCCAGCTTAAGGGAACTATTAACGG	
	AAAGTCTTTCACTGTTGAAGGAGAAGGAG	
	AAGGAAACTCTCACGAAGGATCTCATAAG	
	GGAAAGTACGTTTGCACTTCTGGAAAGCTT	
	CCAATGTCTTGGGCTGCTCTTGGAACTACT	
	TTCGGATATGGAATGAAGTATTACACTAAGT	
	ACCCATCTGGACTTAAGgtaagtttaaacatatatatact	
	aactaaccctgattatttaaattttcagAACTGGTTCCGCGA	
	AGTTATGCCAGGAGGATTCACTTATGATCG	
	CCATATCCAGTACAAGGGAGATGGATCTAT	
	CCATGCTAAGCATCAGCATTTCATGAAGAA	
	TGGAACTTACCACAACATCGTTGAATTCAC	
	TGGACAAGATTTCAAGgtaagtttaaacagttcggtact	
	aactaaccatacatatttaaattttcagGAAAACTCCCCAG	
	TTCTTACCGGAGATATGAACGTTTCCCTTCC	
	AAACGAAGTTCCACAAATCCCACGTGATGA	
	TGGAGTTGAGTGCCCAGTTACCCTTCTTTA	
	CCCACTTCTTTCCGATAAGTCCAAGTACGT	
	TGAGGCTCATCAGTACACTATCTGCAAGCC	
	ACTTCATAACCAACCAGCTCCAGATGTTCC	
	ATACCATTGGATCCGCAAGCAATACACTCA	
	ATCTAAGGATGATGCTGAGGAGCGCGATCA	
	CATCTGCCAATCTGAGACTCTTGAGGCTCA	
	CCTTAAGGGAATGGATGAGCTTTACAAGTA	
	ATAActaccatctctg-3’	
cosa-1^{R163A}	5’-ATTGCAGTTAGAATTCTCAA-3’	F 5’-caaaatgttctcttcgaggagt-3’
	5’-cgcgtatacATCATGATAACTGTGAAATTTGC	R 5’-cggagcatcgttgaaattcg-3’
	TGCTAATCTGAATTGCAGTTAGAATagcCAA	Xho I
	CGGAATCTGTCGTTCCATATTCGAcTCgAGA	WT: 286 bp
	TTTTCCCATTCTCTTTTCTTCCGTTGAATCG	Mutant: 117 bp＆163 bp
	ACATTTCTTGTC-3’	
slx-4::AID::	5’-AGACGGGAAGAGCACGTGG-3’	F 5’-CACTCCAACCTGTATCCTTGgt-3’
3×FLAG	5’-GAGAAAGCTGACGGCCCACTTGGAAGG	R 5’-gaatactggatgtgctccaag-3’
	ATTGGAAAAGGAAAACTGGTGAAATTACT	WT: 398 bp
	GGAAATGCTGAAAATCACGTACCAACTTCC	Mutant: 620 bp
	ACAGAAGACGGGAAGAGCACGTGGCGGCT	
	ACAAAAGAAGACTAGGAGGCTCAGGAatgcc	
	taaagatccagccaaacctccggccaaggcacaagttgtgggatgg	
	ccaccggtgagatcataccggaagaacgtgatggtttcctgccaaaa	
	atcaagcggtggcccggaggcggcggcgttcgtgaagGGATC	

	GGACTATAAAGATCACGACGGAGATTACAA<br>GGACCATGATATCGACTACAAGGACGACGA<br>CGACAAGGGATAAcaattttttccatgcaattttatgaagag<br>ataattaggaatataatatgttttcacggaaaatcagagaaattcaaaaa<br>atacaaaaaaaaattagaattgagtttcgttaattcctgatctagttctgta<br>ag-3’<br>5’-GGAGGCTCAGGAatgcctaaagatccagccaaacctc<br>cggccaaggcacaagttgtgggatggccaccggtgagatcataccg<br>gaagaacgtgatggtttcctgccaaaaatcaagcggtggcccggag<br>gcggcggcgttcgtgaagGGATCGGACTATAAAGAT<br>CACGACGGAGATTACAAGGACCATGATATC<br>GACTACAAGGACGACGACGACAAGGGA-3’	
slx-4$^{\triangle 379\text{-}402}$	5’-GTAGAGTGTACTGATTCATG-3’<br>5’-GATCCTGTAGAAATAGAGCCAACTGCGT<br>CTCATAGCGACCCAATCGAACTTGATAAAT<br>CTGTGAACATCATGGAAGATAAACCACAcG<br>AATCtGTtCACTCTACTCCACAGAAACCAAC<br>TACTAATGTGACAATGGATTCATTCGATGAA<br>TGGAGCAATCAACCGTCTACAAATCTTCCA<br>ACTACATCCAATGTCATTACTCCAATCCGCA<br>ACATTAC-3’<br>5’-CTTGATAAATCTGTGAACATCATGGAAGA<br>TAAACCACAcGAATCtGTtCACTCTACTCCAC<br>AGAAACCAACTACTAATGTGACAATGGATT<br>CATTCGATGAATGGAGCAATCAACCG-3’;	F 5’-GATTCTGTAGTAGCTGATCCT-3’<br>R 5’-GTAATGTTGCGGATTGGAGTA-3’<br>WT: 305 bp<br>Mutant: 233 bp
slx-4::3×FLAG::GFP nanobody	5’-AGACGGGAAGAGCACGTGG-3’<br>5’-GAGAAAGCTGACGGCCCACTTGGAAGG<br>ATTGGAAAAGGAAAACTGGTGAAATTACT<br>GGAAATGCTGAAAATCACGTACCAACTTCC<br>ACAGAAGACGGGAAGAGCACGTGGCGGCT<br>ACAAAAGAAGACTAGGATCGGACTATAAA<br>GATCACGACGGAGATTACAAGGACCATGAT<br>ATCGACTACAAGGACGACGACGACAAGGG<br>CTCAGATCAAGTCCAACTGGTGGAGTCTGG<br>TGGCGCTTTGGTGCAGCCAGGTGGCTCTCT<br>GCGTTTGTCCTGTGCCGCTTCTGGCTTCCC<br>AGTGAACCGCTATTCCATGCGCTGGTATCG<br>CCAGGCTCCAGGCAAAGAGCGTGAGTGGG<br>TAGCCGGTATGTCCAGCGCGGGTGATCGTA<br>GCTCCTATGAAGACTCCGTGAAGGGCCGTT<br>TCACCATCAGCCGTGACGATGCCCGTAACA<br>CGGTGTATCTGCAAATGAACAGCTTGAAAC<br>CTGAAGATACGGCCGTGTATTACTGTAATGT<br>GAACGTGGGCTTCGAGTATTGGGGCCAAG	F 5’-CACTCCAACCTGTATCCTTGgt-3’<br>R 5’-gaatactggatgtgctccaag-3’<br>WT: 398 bp<br>Mutant: 824 bp

	GCACCCAGGTCACCGTCTCCAGCTAAcaatttt<br>tccatgcaattttatgaagagataattaggaatataatatgttttcacgga<br>aaatcagagaaattcaaaaaatacaaaaaaaaaattagaattgagtttcg<br>ttaattcctgatctagttctgtaag-3’；<br>5’-GGATCGGACTATAAAGATCACGACGGAG<br>ATTACAAGGACCATGATATCGACTACAAGG<br>ACGACGACGACAAGGGCTCAGATCAAGTC<br>CAACTGGTGGAGTCTGGTGGCGCTTTGGTG<br>CAGCCAGGTGGCTCTCTGCGTTTGTCCTGT<br>GCCGCTTCTGGCTTCCCAGTGAACCGCTAT<br>TCCATGCGCTGGTATCGCCAGGCTCCAGGC<br>AAAGAGCGTGAGTGGGTAGCCGGTATGTC<br>CAGCGCGGGTGATCGTAGCTCCTATGAAGA<br>CTCCGTGAAGGGCCGTTTCACCATCAGCCG<br>TGACGATGCCCGTAACACGGTGTATCTGCA<br>AATGAACAGCTTGAAACCTGAAGATACGG<br>CCGTGTATTACTGTAATGTGAACGTGGGCT<br>TCGAGTATTGGGGCCAAGGCACCCAGGTC<br>ACCGTCTCCAGC-3’	
spo-11	5’-gcacgggtcccgcagcgaat-3’<br>5’-agtactgagactgagaagtg-3’<br>5’-gtcgattggccagagaggcaaagtgaggagaaacactattggaa<br>atggagagacgcagacactctgcatgtctgcctgcattggaaacggg<br>ccattgcggcggagaggacaaatgaatgggaaaagagacaagaaa<br>agaaggaatagtggaaaatacaagaagactacggtagatatagacca<br>tatcagagcttcattatgactatttgaataaagtatttgaaggaaatttaat<br>atagcaaaaatatttcagcgcaacaacatttaatgctagtttaagaaaaa<br>aatgaatactatacataaattttatattctataaaaatcttcttcgactgcg<br>acgaaatgaatggtgcagtcattcgcttcaaaattgcatttaagctgtgg<br>tttttcggcagggattgagaaaaaccgagttatatttaagaaatgcatttt<br>aaaatattttttgaagaaaaatgtcaatttaagaatatagaaaagaagat<br>cgaacattgagactcgtcggcgccgagtattaaaaatctgacgaaaat<br>tacagtactactccggtggggggagaaacaacattaaataaatagaa<br>aacaacacaagttatcttatcttatcacaatatcatcgagtgcataaagc<br>taatggaatggggagatgttttacattgtttagagctcagagtgctcctc<br>ctcaatcgttgtttcttcagcggcttctggctctggttcagcctcttcaata<br>tgctgttcggtttcaacttgagcatcttgggaaacatcgagcgattggc<br>gaaggacagcttcgattctatcagcaaaaccaacttgatcttgaagtga<br>gaatccagaacgaagagtggcagtttcgaagagaagcttggcagta<br>gaagcagcggtggtatcttcttcagaagcagtgactctcttgagaagct<br>ccttgataactggatggcgtgggttaatctcgaatgtctttttctgagtag<br>catagaagtcttgagttggatcctttgcc-3’	F 5’-gtcgattggccagagaggc-3’<br>R 5’ -ggatggtctggaaacatggag-3’<br>WT: 2900 bp<br>Mutant: 1058 bp

Table S3 Strains used in this study

Strain #	Strains	Source
sYH_0007	N2	Caenorhabditis Genetics Center
sYH_0059	*GFP::msh-5 IV*	Janisiw et al, 2018
sYH_0067	*GFP::cosa-1 (AV630)* *meIs8 [pie-1p::GFP::cosa-1 + unc-119 (+)] II*	Yokoo et al., 2012
sYH_0087	*zhp-3::GFP [pie-1p::zhp-3::gfp] IV*	Bhalla et al, 2008
sYH_0153	*slx-4/him-18 (tm2181)/qC1* *[dpy-19(e1259) glp-1(q339) qIs26] III (CV98)*	Caenorhabditis Genetics Center
sYH_0369	*cosa-1::3×HA::TurboID III*	Yang et al., 2024
sYH_0373	*slx-4::AID::3×FLAG III*	This study
sYH_0431	*cosa-1::3×HA::TurboID III* *slx-4::AID::3×FLAG III*	This study
sYH_0715	*slx-4*$^{\triangle 379\text{-}402}$*::AID::3×FLAG III*	This study
sYH_0739	*slx-4*$^{\triangle 379\text{-}402}$*::AID::3×FLAG III; zhp-3::GFP IV*	This study
sYH_0740	*spo-11/nT1 IV*	This study
sYH_0800	*slx-4*$^{\triangle 379\text{-}402}$*::3×FLAG ::GFP nanobody III*	This study
sYH_0801	*GFP::cosa-1 II;* *slx-4*$^{\triangle 379\text{-}402}$*::3×FLAG::GFP nanobody III;*	This study
sYH_0840	*slx-4*$^{\triangle 379\text{-}402}$*::AID::3×FLAG III; spo-11/nT1 IV*	This study
sYH_0866	*cosa-1::mBaojin III*	This study
sYH_0889	*cosa-1*^{R163A}*::mBaojin III*	This study
sYH_0892	*cosa-1::mBaojin III slx-4::AID::3×FLAG III*	This study
sYH_0896	*slx-4::AID::3×FLAG III; GFP::msh-5 IV*	This study
sYH_0898	*cosa-1::mBaojin III* *slx-4*$^{\triangle 379\text{-}402}$*::3×FLAG::GFP nanobody III*	This study
sYH_0899	*slx-4::3×FLAG ::GFP nanobody III*	This study
sYH_0900	*slx-4*$^{\triangle 379\text{-}402}$*::3×FLAG::GFP nanobody III; zhp-3::GFP IV*	This study

wsYH0103	*slx-4::AID::3×FLAG III; TIR1 IV*	This study
wsYH0143	*slx-4*$^{\triangle 379\text{-}402}$*::AID::3×FLAG III; GFP::msh-5 IV*	This study
wsYH0145	*cosa-1::mBaojin III*<br>*slx-4*$^{\triangle 379\text{-}402}$*::AID::3×FLAG III*	This study
wsYH0146	*cosa-1::3×HA::TurboID III*<br>*slx-4*$^{\triangle 379\text{-}402}$*::AID::3×FLAG III*	This study
wsYH0149	*GFP::cosa-1 II;*<br>*slx-4::3×FLAG::GFP nanobody III*	This study
wsYH0152	*cosa-1*^{R163A}*::3×HA::TurboID III*<br>*slx-4::AID::3×FLAG III*	This study
